# Supplementary material for: Neogrisphenol A, a Potential Ovarian Cancer Inhibitor from a New Record Fungus Neohelicosporium griseum
Source: Metabolites. 2023 Mar 16;13(3):435. doi: 10.3390/metabo13030435 (PMC10055829; doi:10.3390/metabo13030435)
Supplement: Supplementary file 1 [file metabolites-13-00435-s001.zip › metabolites-2260186-supplementary final.pdf]

Supplementary Material

Neogrisphenol A, a Potential Ovarian Cancer Inhibitor from a New Record Fungus Neohelicosporium griseum

Li-Juan Zhang [1,2,3,†], Ming-Fei Yang [4,5,6,†], Jian Ma [2,3], Xing-Juan Xiao [1], Xiao-Yan Ma [1], De-Ge Zheng [1], Mei-Yan Han [1], Ming-Lei Xia [1], Ruvishika S. Jayawardena [2,3], Ausana Mapook [2], Yuan-Pin Xiao [1], Ji-Chuan Kang [4,*] and Yong-Zhong Lu [1,4,*]

[1] School of Food and Pharmaceutical Engineering, Guizhou Institute of Technology, Guiyang 550003, China; 6471105010@lamduan.mfu.ac.th (L.-J.Z.); xiaoxingjuanbenjuan@gmail.com (X.-J.X.); 20120065@git.edu.cn (X.-Y.M.); degezheng@gmail.com (D.-G.Z.); meiyanhan04@gmail.com (M.-Y.H.); xml0312xml@gmail.com (M.-L.X.); emaypx@gmail.com (Y.-P.X.)

[2] Center of Excellence in Fungal Research, Mae Fah Luang University, Chiang Rai 57100, Thailand; 6371105007mj@gmail.com (J.M.); ruvishika.jay@mfu.ac.th (R.S.J.); Phung.ausana@gmail.com (A.M.)

[3] School of Science, Mae Fah Luang University, Chiang Rai 57100, Thailand

[4] Engineering and Research Center for Southwest Bio-Pharmaceutical Resources of National Education Ministry of China, Guizhou University, Guiyang 550025, China; mf_yang@126.com

[5] College of Life Sciences, Guizhou University, Guiyang 550025, China

[6] Department of Health Management, Guiyang Healthcare Vocational University, Guiyang 550081, China

* Correspondence: jckang@gzu.edu.cn (J.-C.K.); yzlu@git.edu.cn (Y.-Z.L.); Tel.: +86-058188210723 (Y.-Z.L.)

† These authors equally contributed to this work.

No.	Contents	Pages

Figure S1. ^{1}H NMR spectrum of compound **1** (600 MHz, DMSO)....3
Figure S2. ^{13}C NMR spectrum of compound **1** (150 MHz, DMSO)....3
Figure S3. ^{1}H-^{1}H COSY spectrum of compound **1** (DMSO)....4
Figure S4. HSQC spectrum of compound **1** (DMSO)....4
Figure S5. HMBC spectrum of compound **1** (DMSO)....5
Figure S6. NOESY spectrum of compound **1**(DMSO)....5
Figure S7. ESIMS of compound **1**....6
Figure S8. HR-ESIMS of compound **1**....7
Figure S9. IR (KBr) spectrum of compound **1**....8
Figure S10. ^{1}H NMR spectrum of compound **2** (600 MHz, DMSO)....9
Figure S11. ^{13}C NMR spectrum of compound **2** (150 MHz, DMSO)....9
Figure S12. ^{1}H-^{1}H COSY spectrum of compound **2** (DMSO)....10
Figure S13. HSQC spectrum of compound **2** (DMSO)....10
Figure S14. HMBC spectrum of compound **2** (DMSO)....11
Figure S15. NOESY spectrum of compound **2** (DMSO)....11
Figure S16. ESIMS of compound **2**....12
Figure S17. HR-ESIMS of compound **2**....13
Figure S18. IR (KBr) spectrum of compound **2**....14
Figure S19. ^{1}H NMR spectrum of compound **3** (600 MHz, DMSO)....15
Figure S20. ^{13}C NMR spectrum of compound **3** (150 MHz, DMSO)....15
Figure S21. ^{1}H-^{1}H COSY spectrum of compound **3** (DMSO)....16
Figure S22. HSQC spectrum of compound **3** (DMSO)....16
Figure S23. HMBC spectrum of compound **3** (DMSO)....17
Figure S24. ESIMS of compound **3**....18
Figure S25. HR-ESIMS of compound **3**....19
Figure S26. IR (KBr) spectrum of compound **3**....20
Figure S27. ^{1}H NMR spectrum of compound **4** (600 MHz, DMSO)....21
Figure S28. ^{13}C NMR spectrum of compound **4** (150 MHz, DMSO)....21
Figure S29. ^{1}H-^{1}H COSY spectrum of compound **4** (DMSO)....22
Figure S30. HSQC spectrum of compound **4** (DMSO)....22
Figure S31. HMBC spectrum of compound **4** (DMSO)....23
Figure S32. ESIMS of compound **4**....24
Figure S33. ^{1}H NMR spectrum of compound **5** (600 MHz, DMSO)....25
Figure S34. ^{13}C NMR spectrum of compound **5**(150 MHz, DMSO)....25
Figure S35. ESIMS of compound **5**....26
Figure S36. ^{1}H NMR spectrum of compound **6** (600 MHz, DMSO)....27
Figure S37. ^{13}C NMR spectrum of compound **6** (150 MHz, DMSO)....27
Figure S38. ESIMS of compound **6**....28
Figure S39. ^{1}H NMR spectrum of compound **7** (600 MHz, $CDCl_3$)....29
Figure S40. ^{13}C NMR spectrum of compound **7** (150 MHz, $CDCl_3$)....29
Figure S41. Optimized geometries of isomers of **1** and **2** at B3LYP/6-31G(d) level....30
Table. S1. Optimized cartesian coordinates of conformers of **1** at B3LYP/6-31G(d) level....31
Table S2. The results of the DP4^{+} analysis of **1**....32
Table. S3. Optimized cartesian coordinates of conformers of **2** at B3LYP/6-31G(d) level....33
Table S4. The results of the DP4^{+} analysis of **2**....34

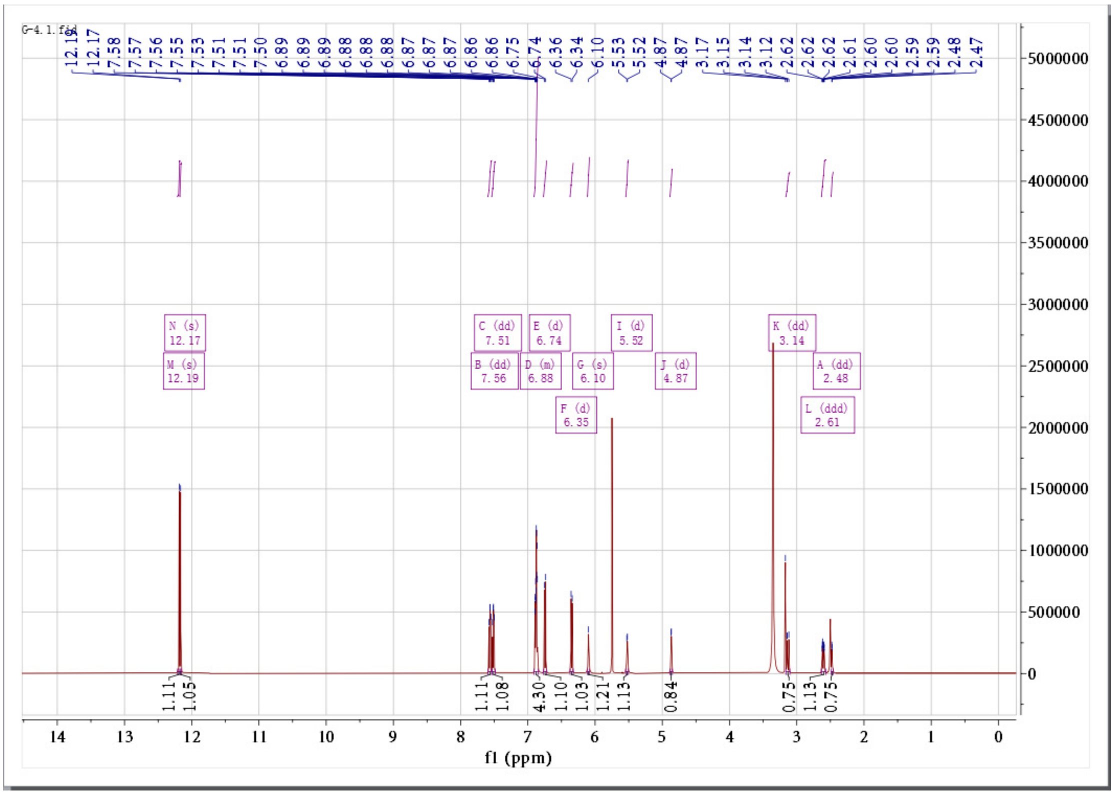

Figure S1. ^{1}H NMR spectrum of compound **1** (600 MHz, DMSO)

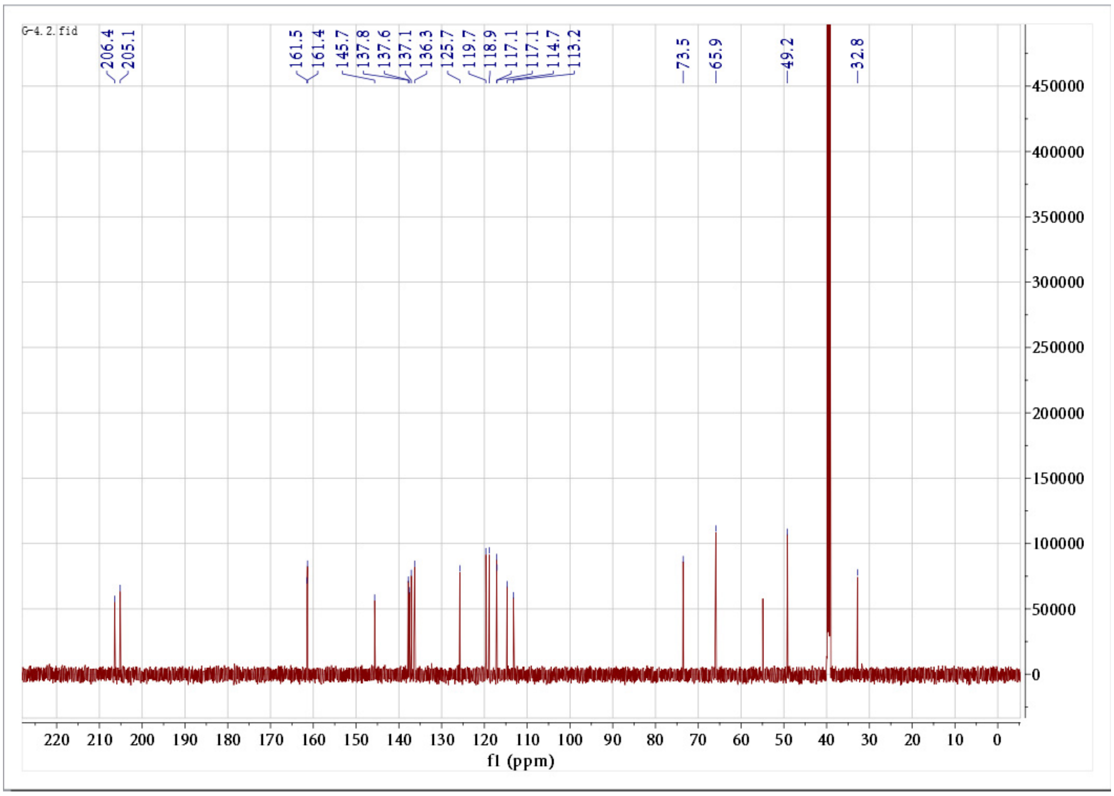

Figure S2. ^{13}C NMR spectrum of compound **1** (150 MHz, DMSO)

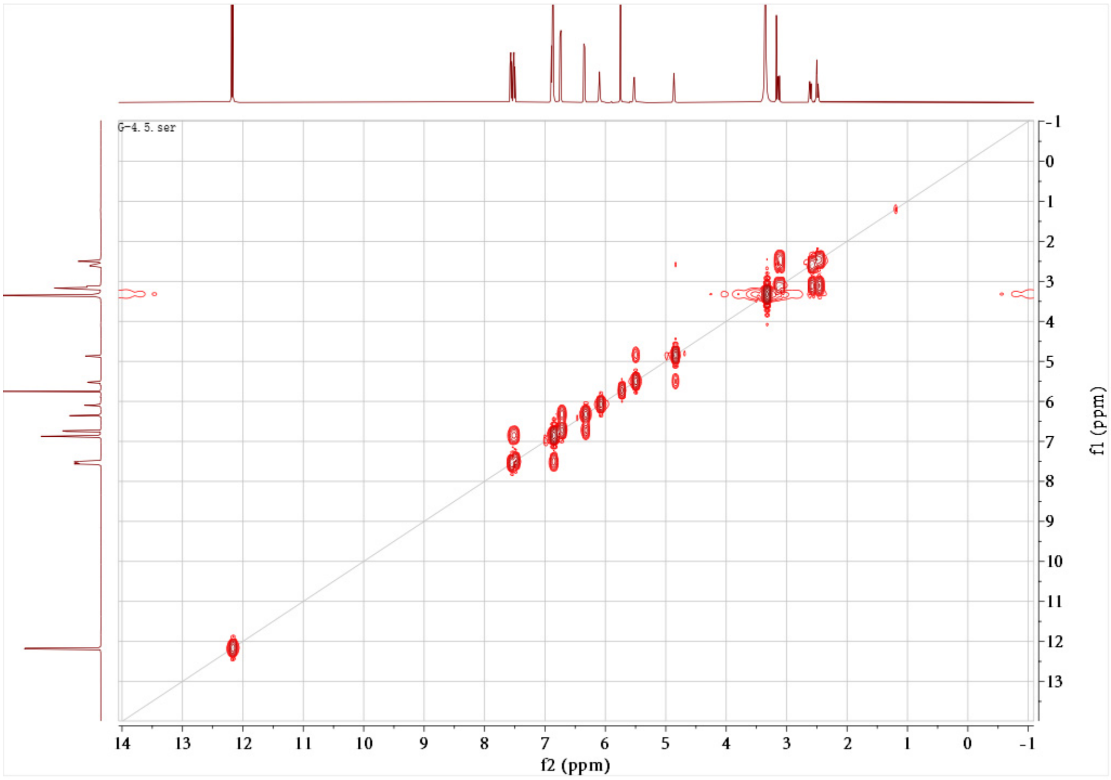

Figure S3. ^{1}H-^{1}H COSY spectrum of compound **1** (DMSO)

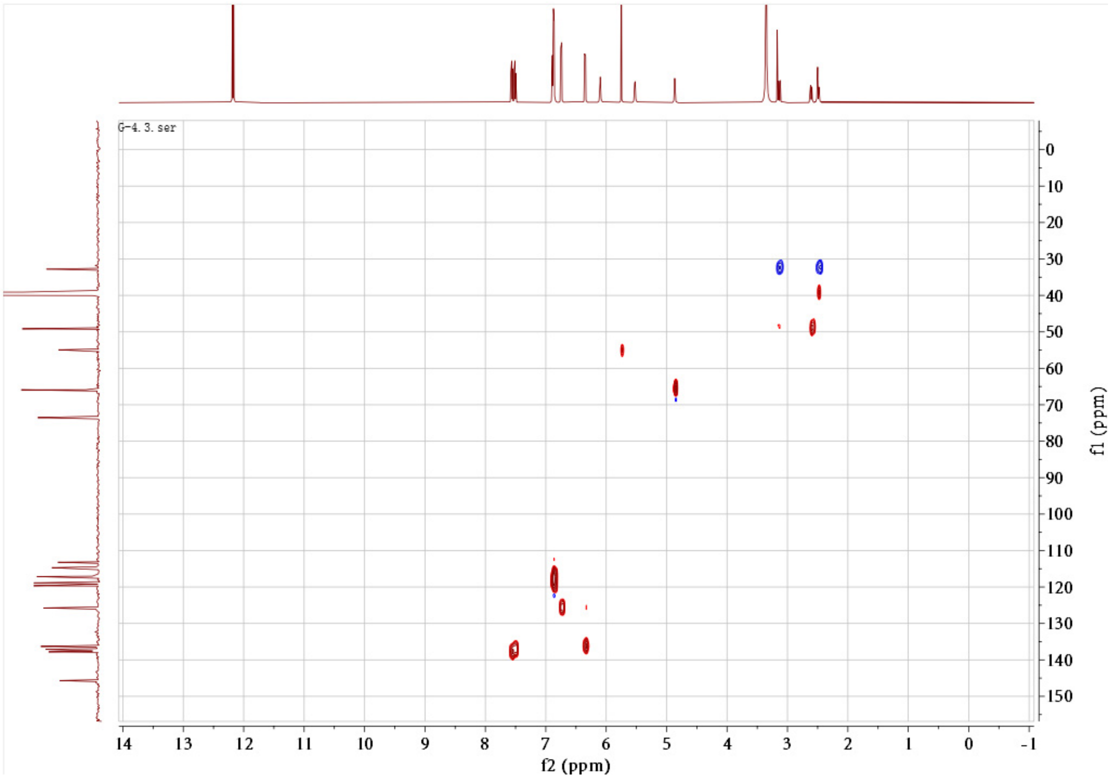

Figure S4. HSQC spectrum of compound **1** (DMSO)

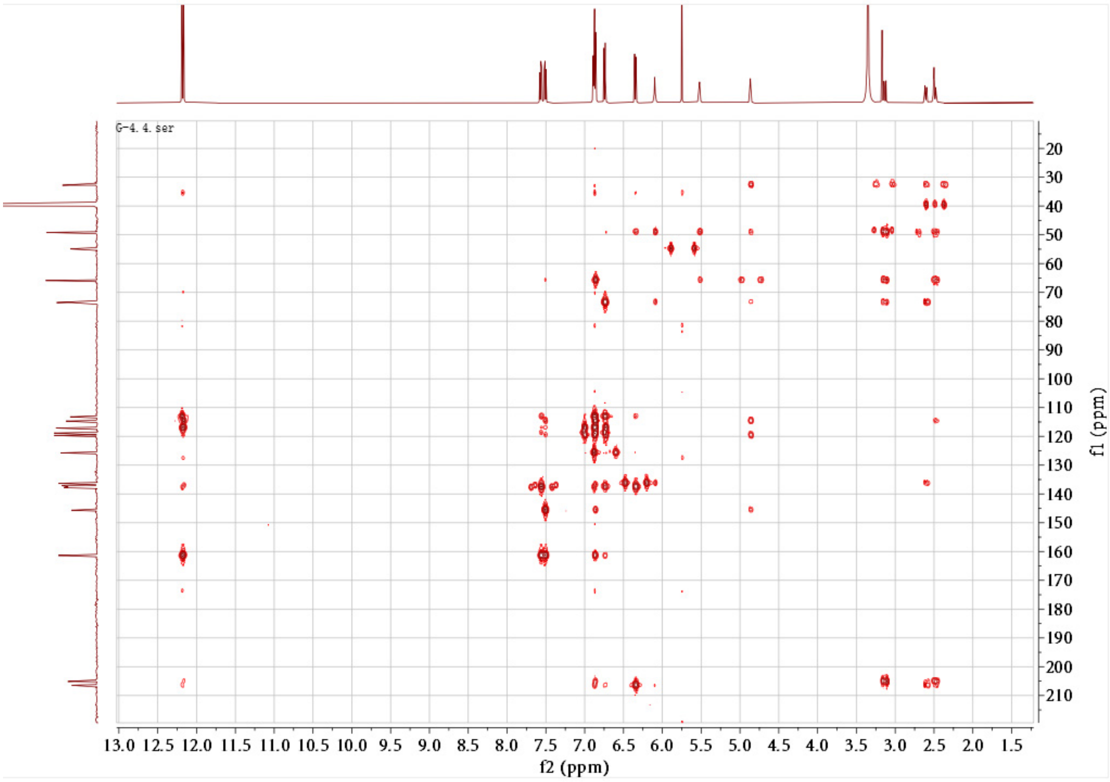

Figure S5. HMBC spectrum of compound **1** (DMSO)

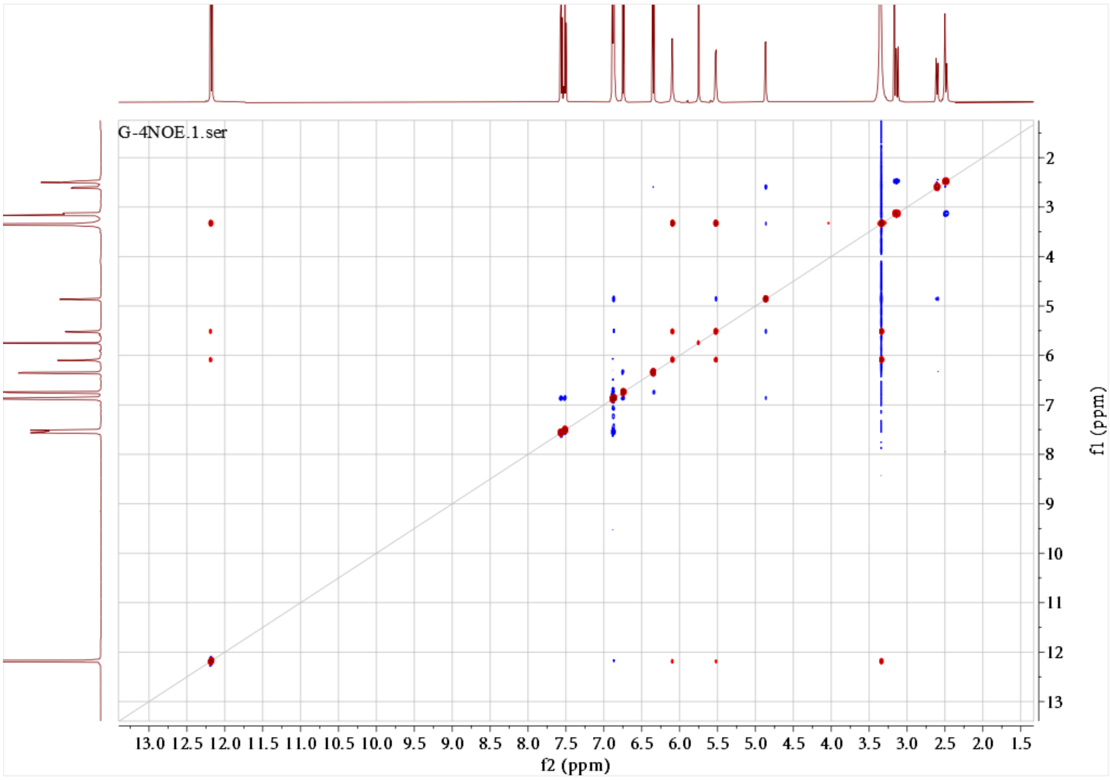

Figure S6. NOESY spectrum of compound **1** (DMSO)

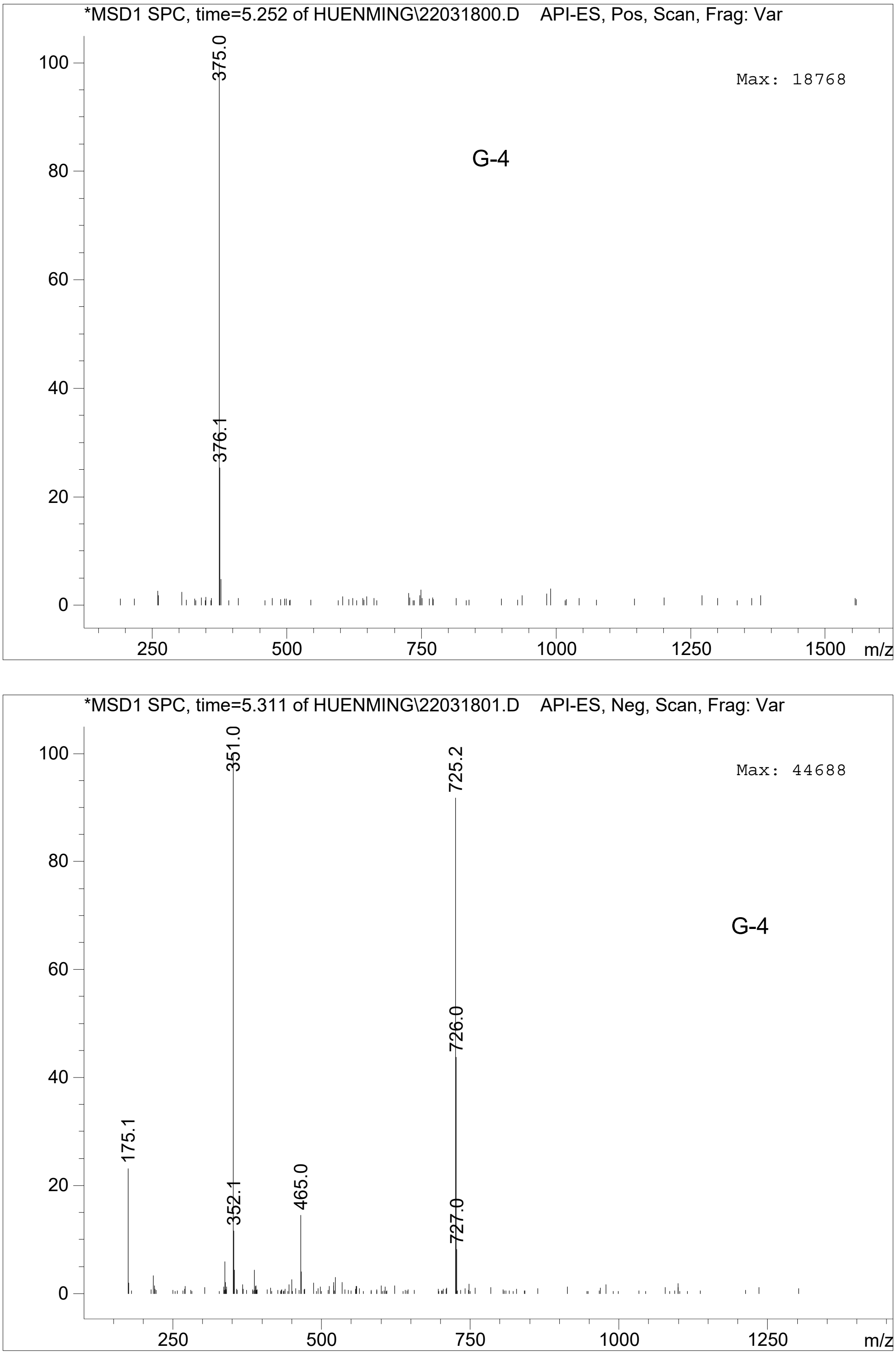

Figure S7. ESIMS of compound **1**

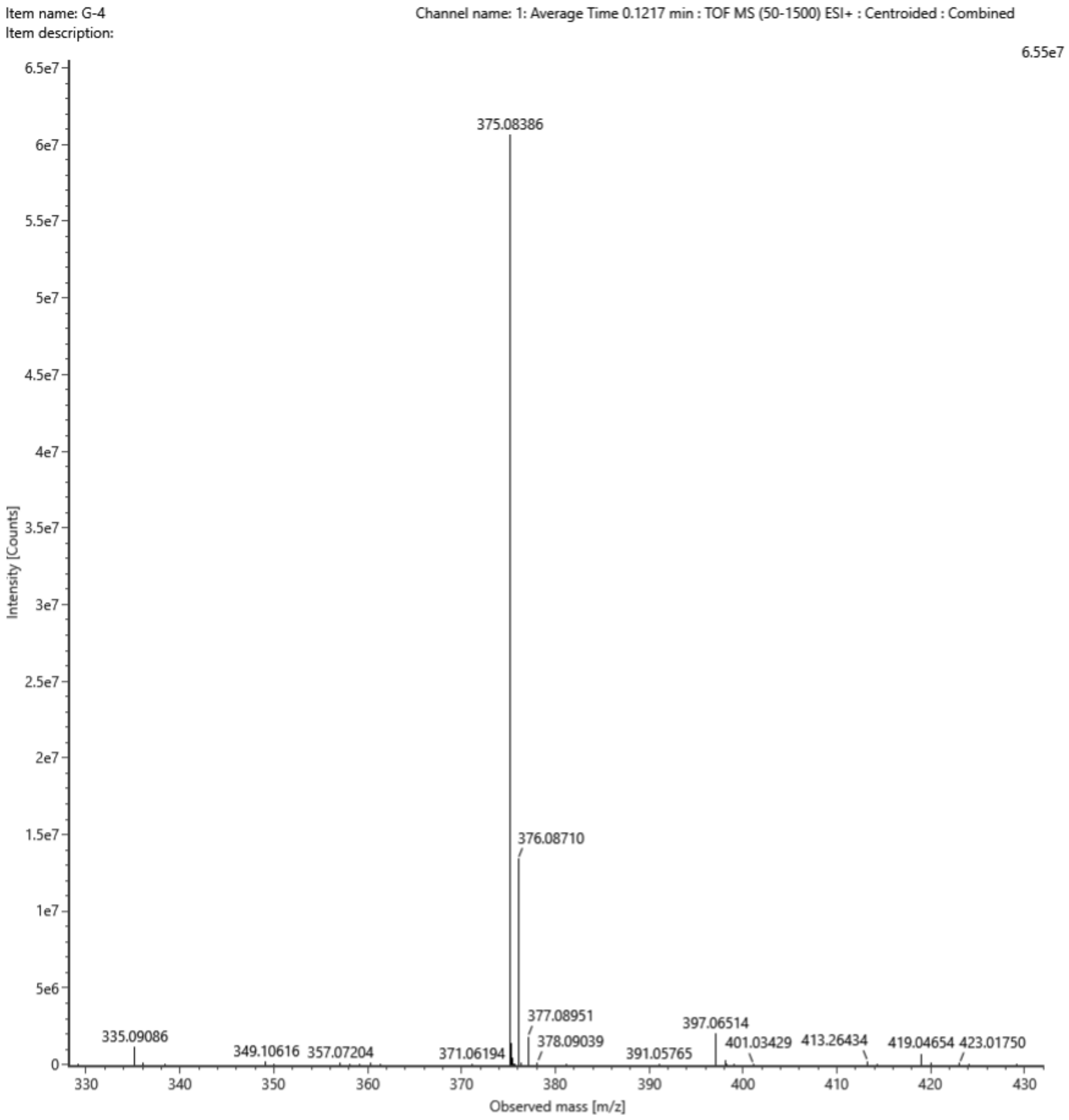

Add:Na+

Composition	i-FIT Confidence (%)	Predicted m/z	m/z error (PPM)
$C_{20}H_{16}O_6Na$	100.000000	375.083909	-0.131856

Figure S8. HR-ESIMS of compound **1**

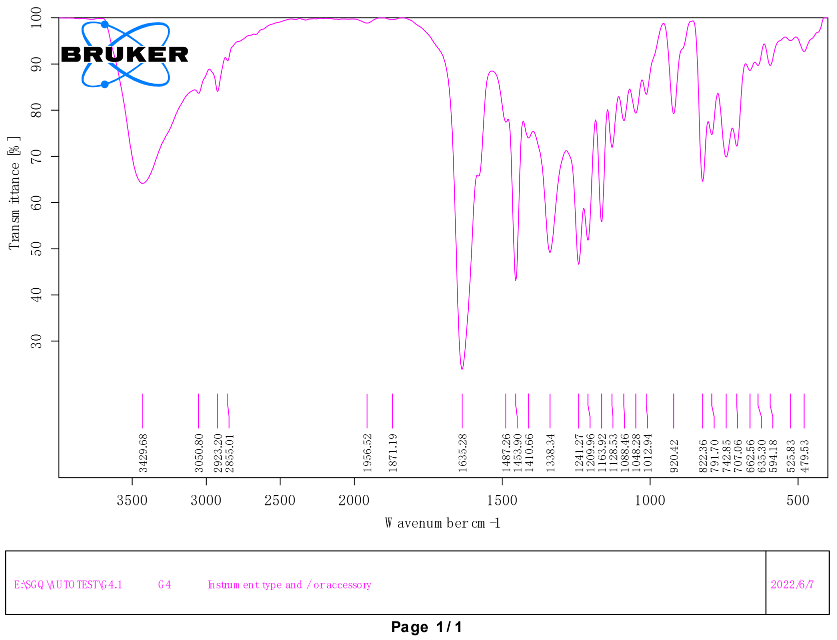

Figure S9. IR (KBr) spectrum of compound **1**

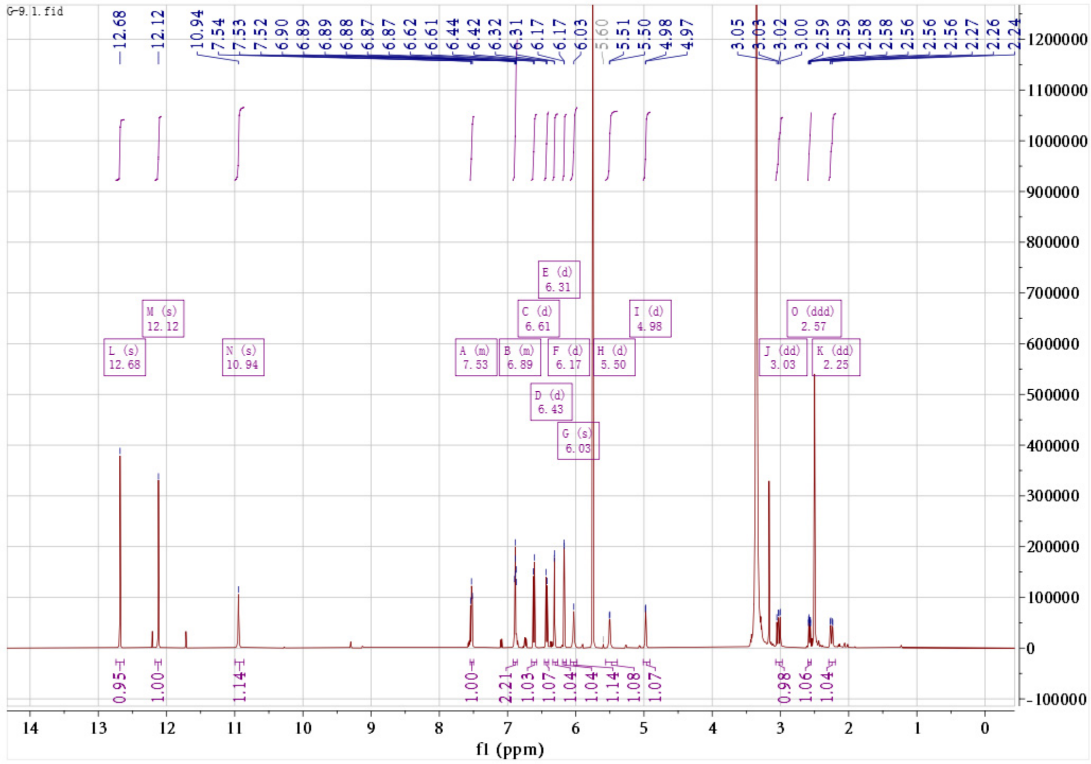

Figure S10. ^{1}H NMR spectrum of compound **2** (600 MHz, DMSO)

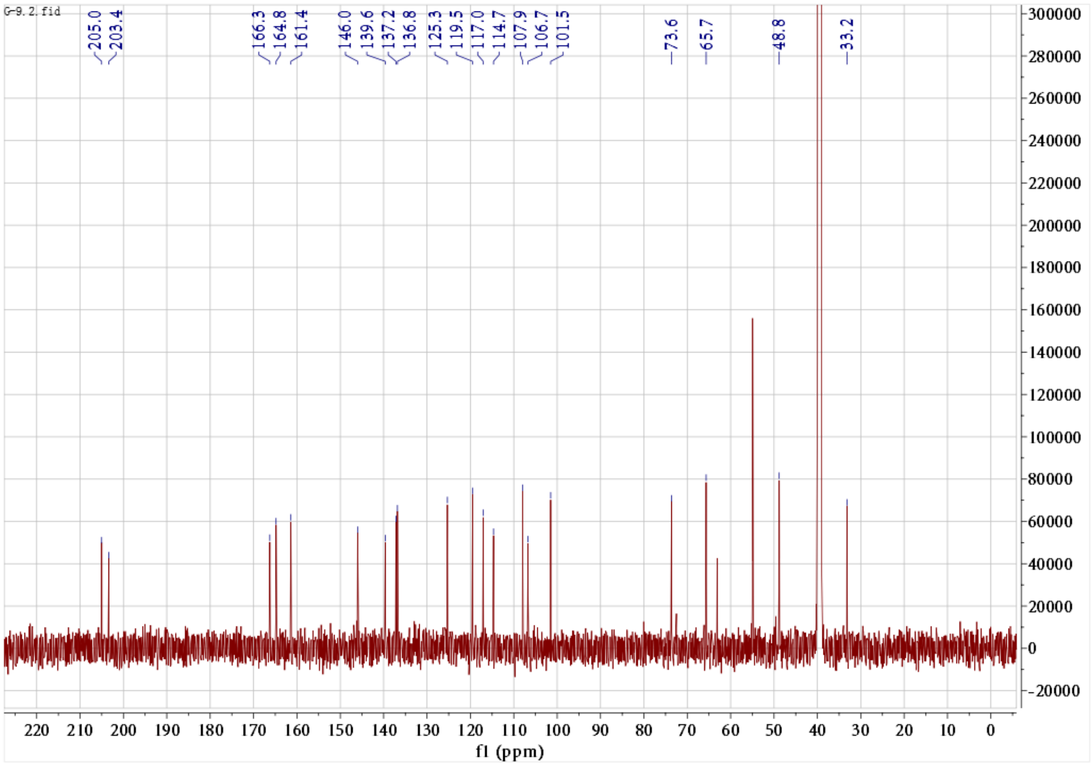

Figure S11. ^{13}C NMR spectrum of compound **2** (150 MHz, DMSO)

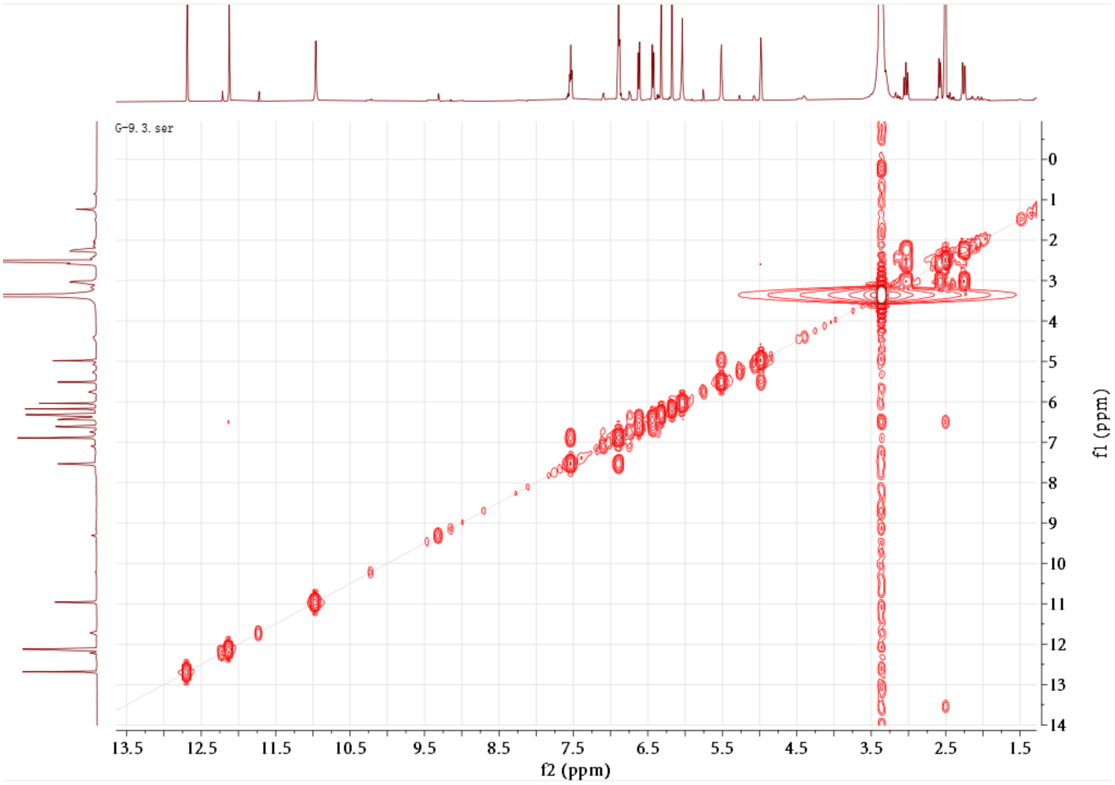

Figure S12. ^{1}H-^{1}H COSY spectrum of compound **2** (DMSO)

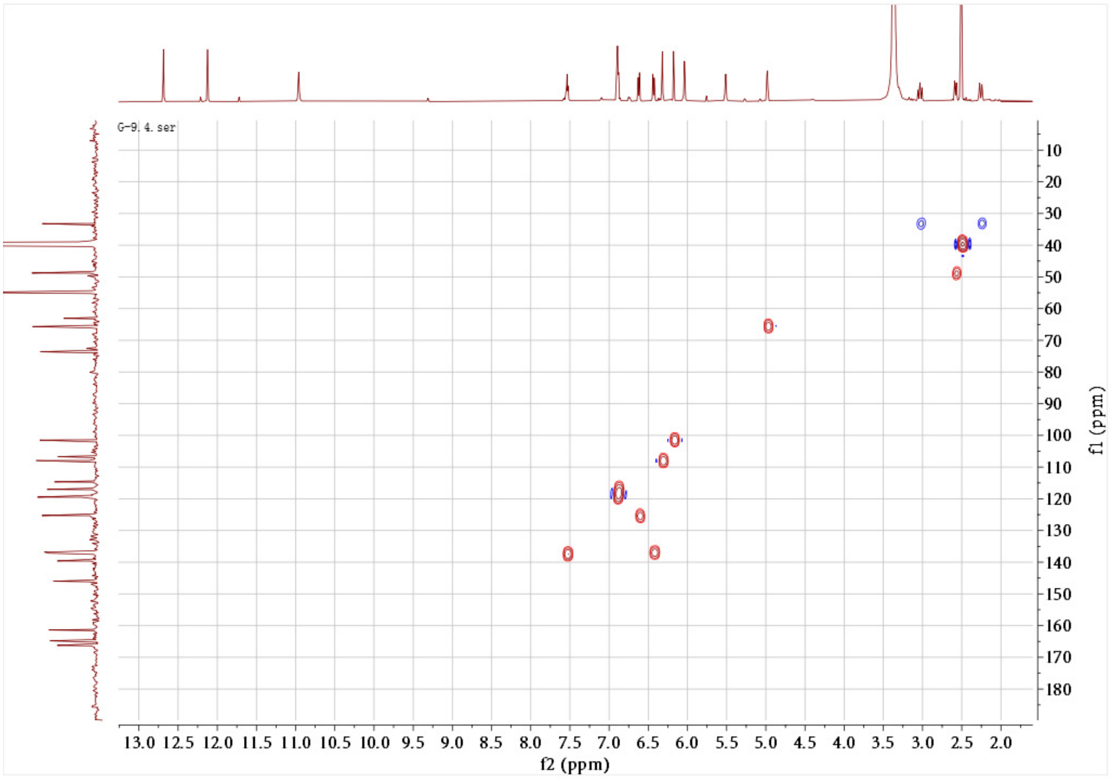

Figure S13. HSQC spectrum of compound **2** (DMSO)

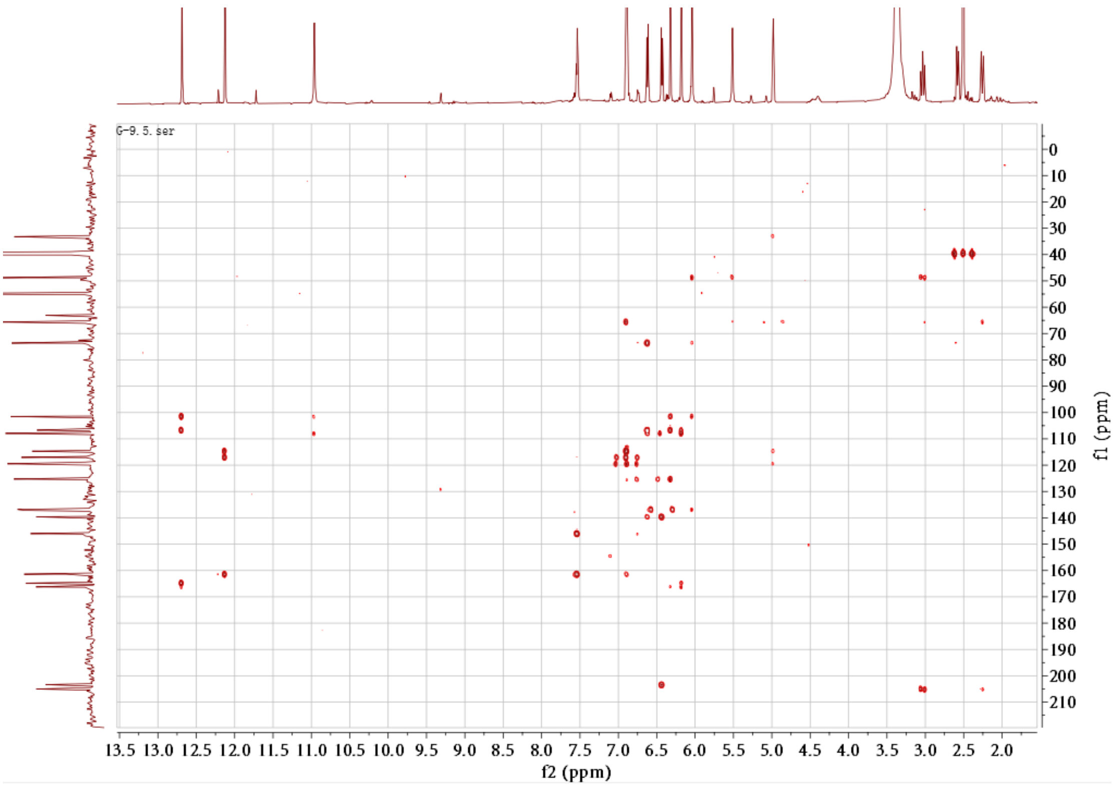

Figure S14. HMBC spectrum of compound **2** (DMSO)

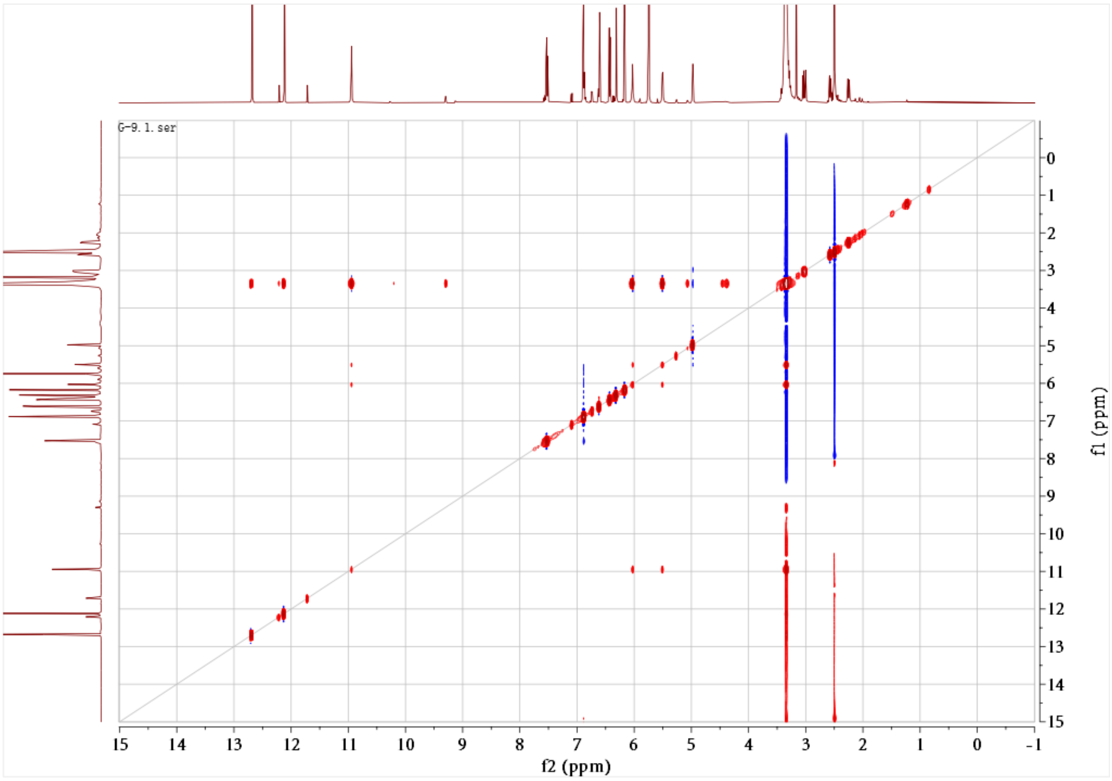

Figure S15. NOESY spectrum of compound **2** (DMSO)

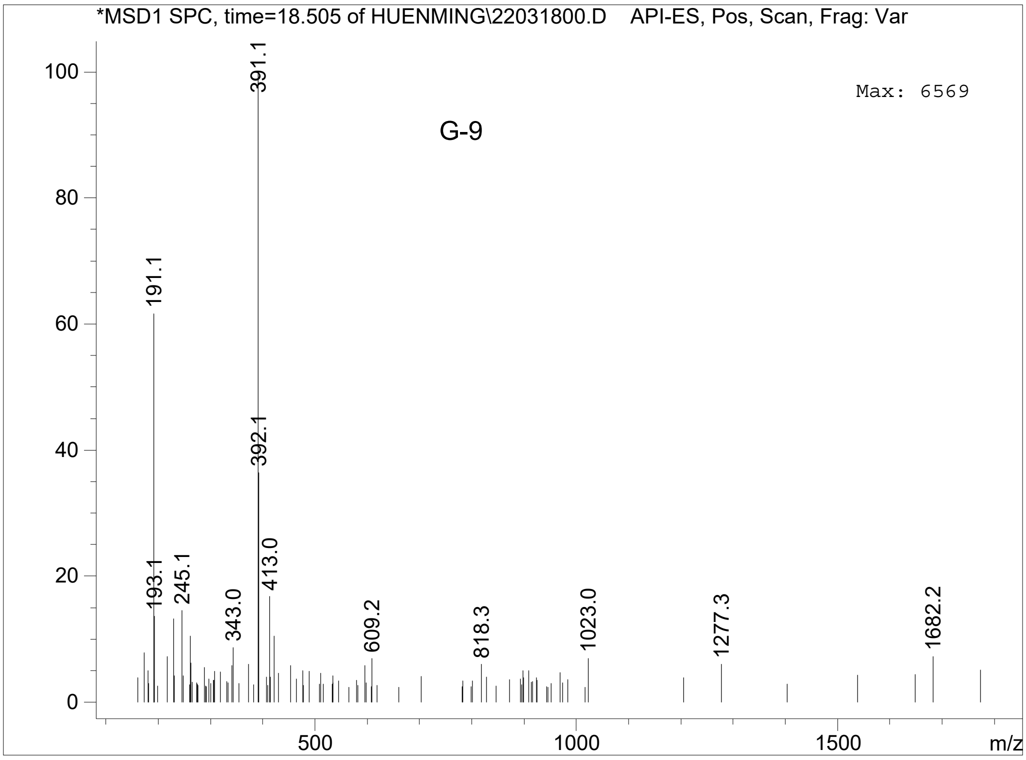

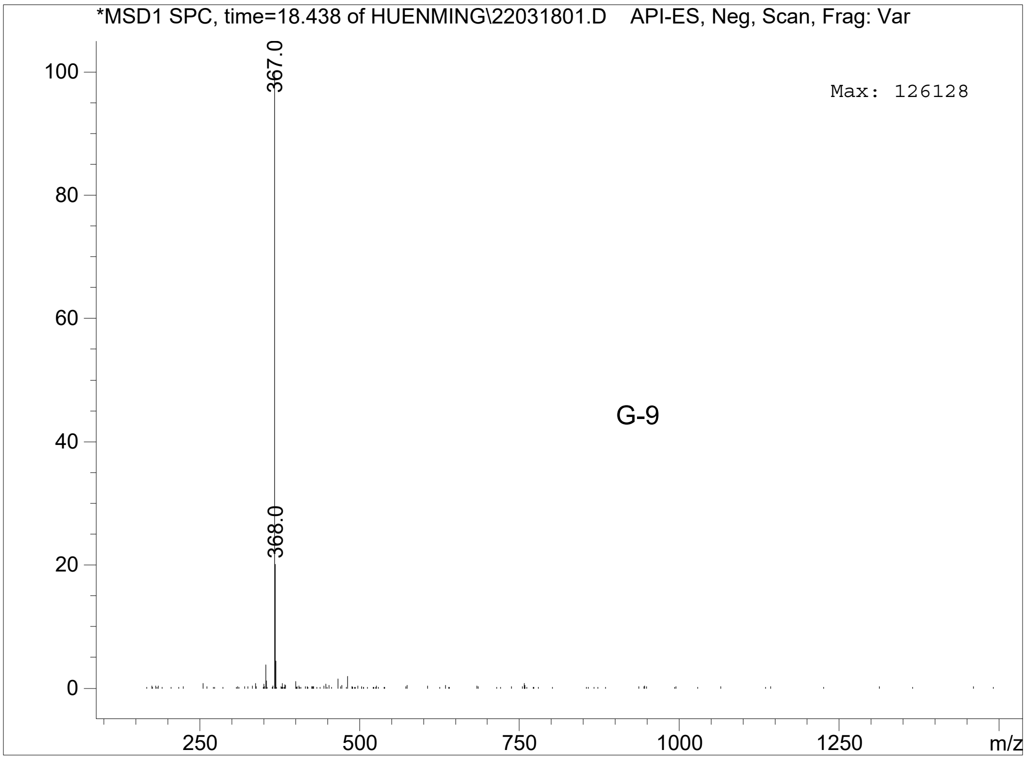

Figure S16. ESIMS of compound **2**

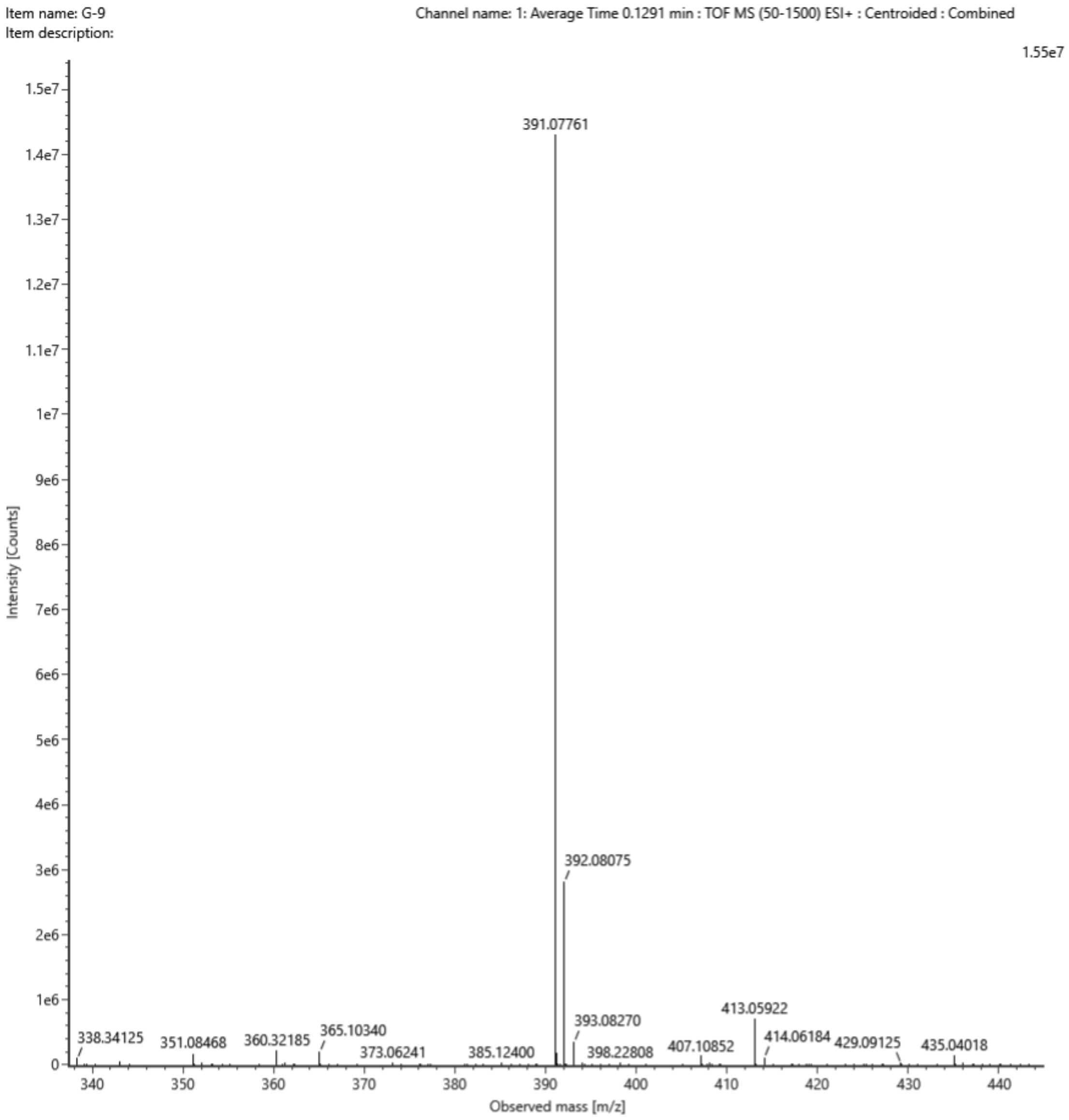

Add:Na+

Composition	i-FIT Confidence (%)	Predicted m/z	m/z error (PPM)
$C_{20}H_{16}O_7Na$	100.000000	391.078824	-3.112122

Figure S17. HR-ESIMS of compound **2**

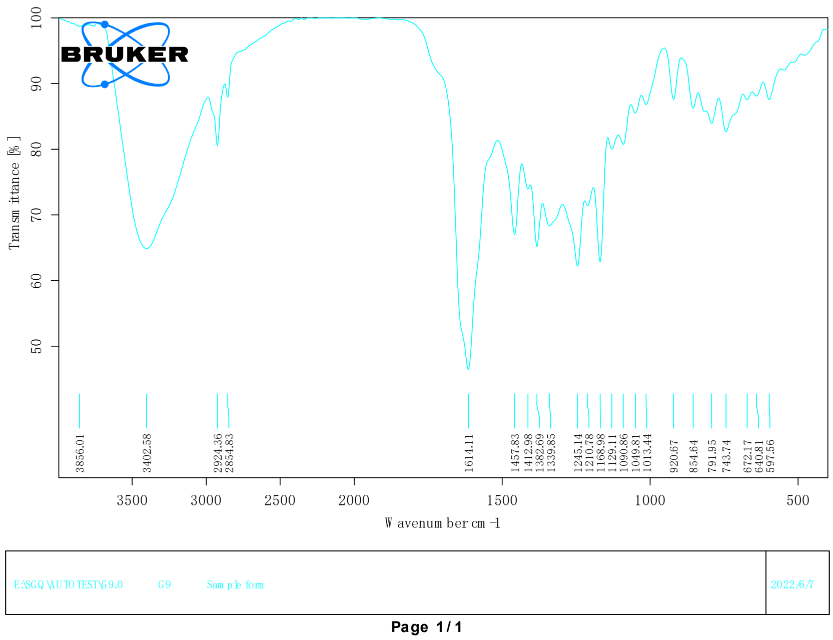

Figure S18. IR (KBr) spectrum of compound **2**

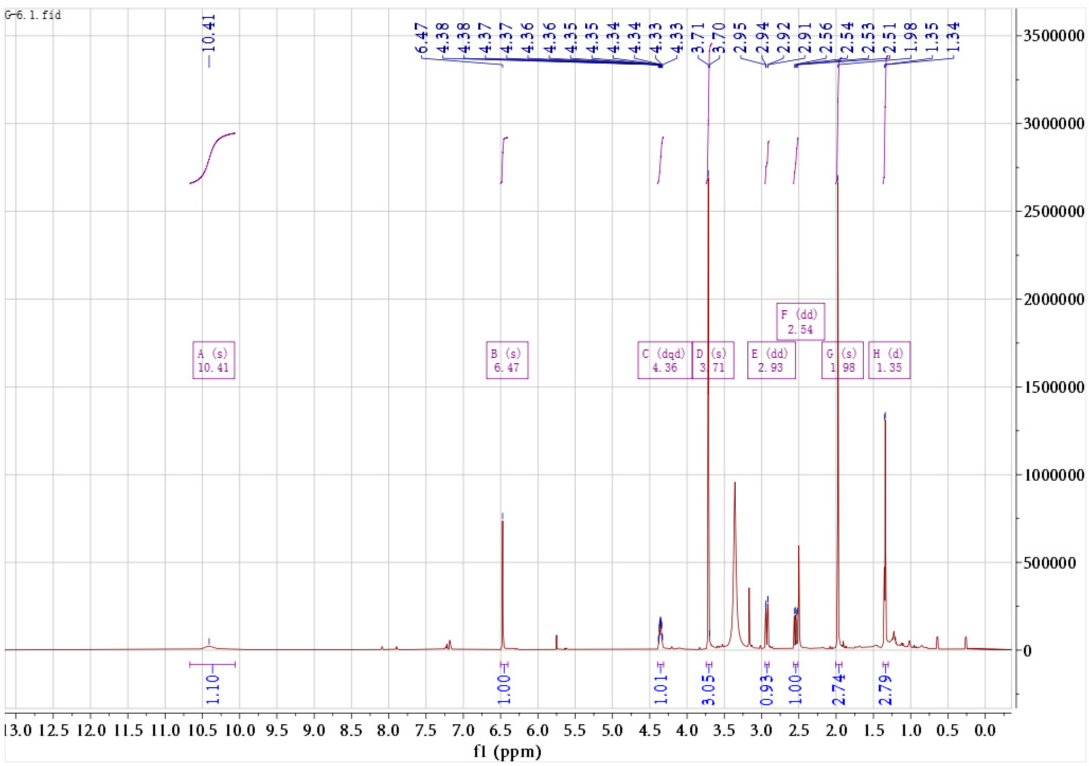

Figure S19. ^{1}H NMR spectrum of compound **3** (600 MHz, DMSO)

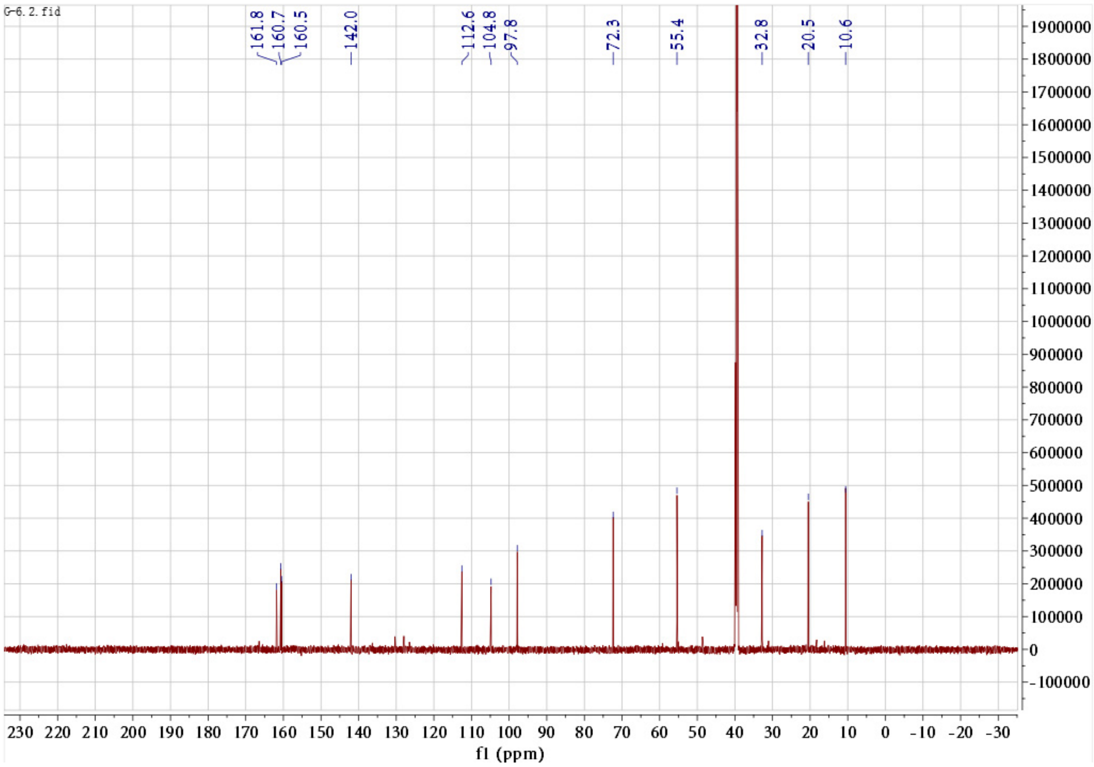

Figure S20. ^{13}C NMR spectrum of compound **3** (150 MHz, DMSO)

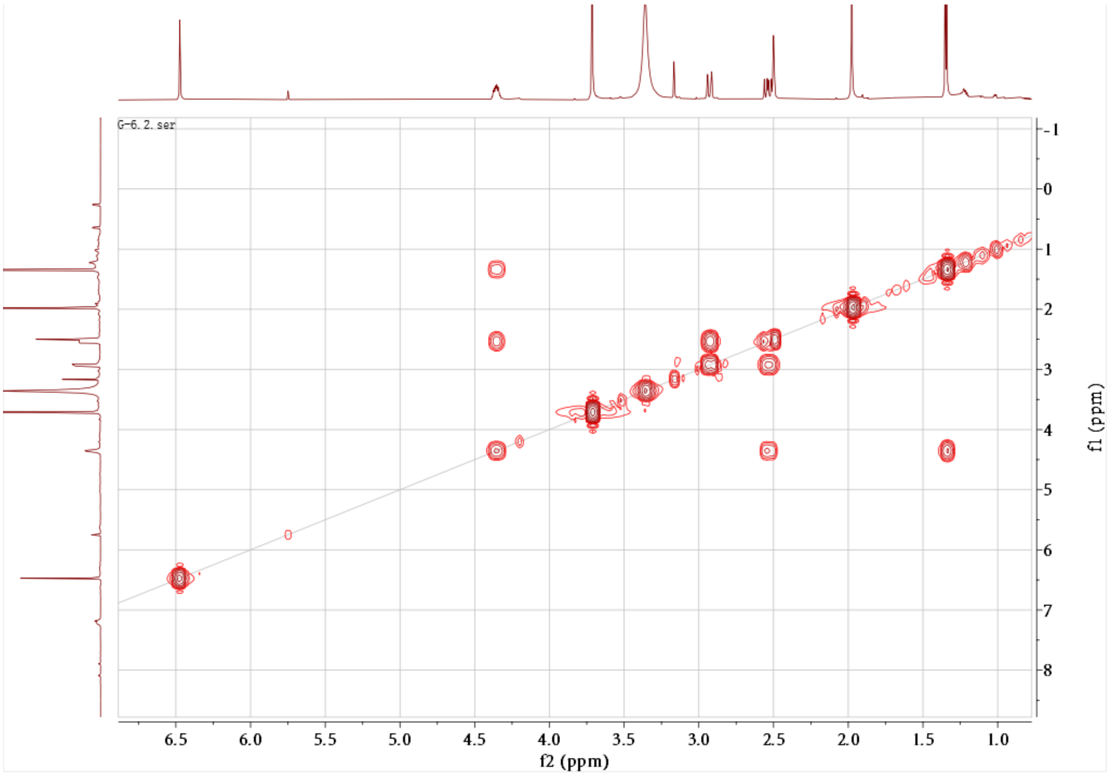

Figure S21. ^{1}H-^{1}H COSY spectrum of compound **3** (DMSO)

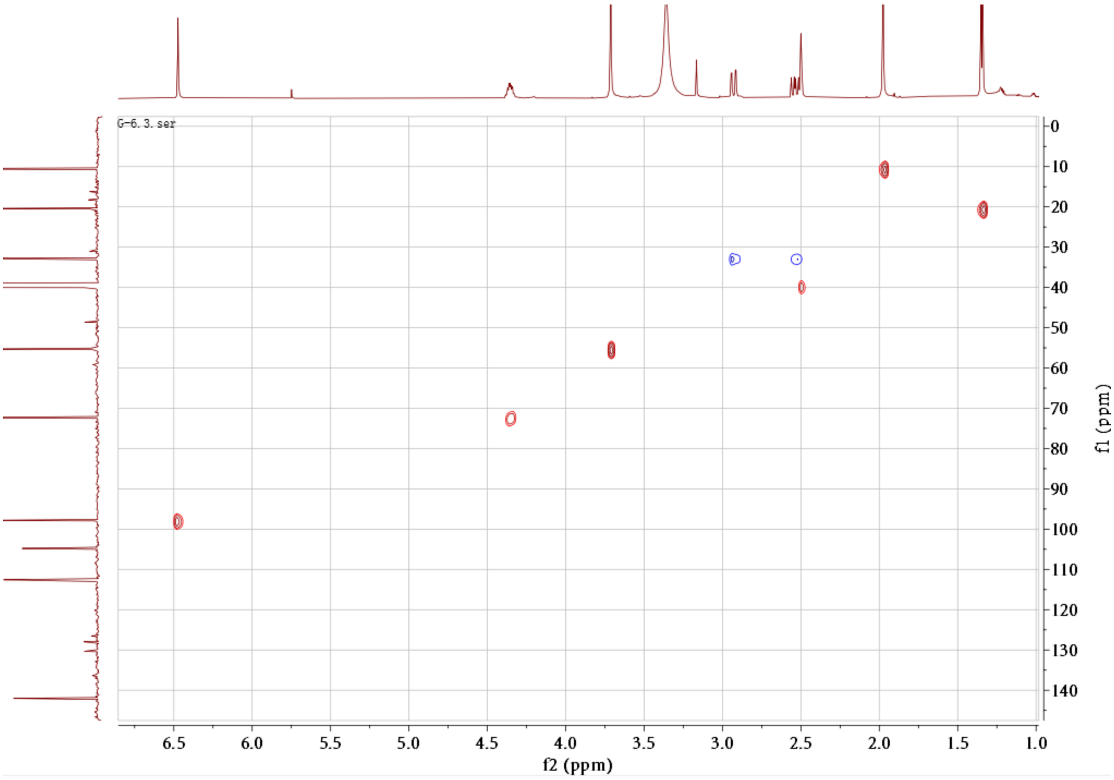

Figure S22. HSQC spectrum of compound **3** (DMSO)

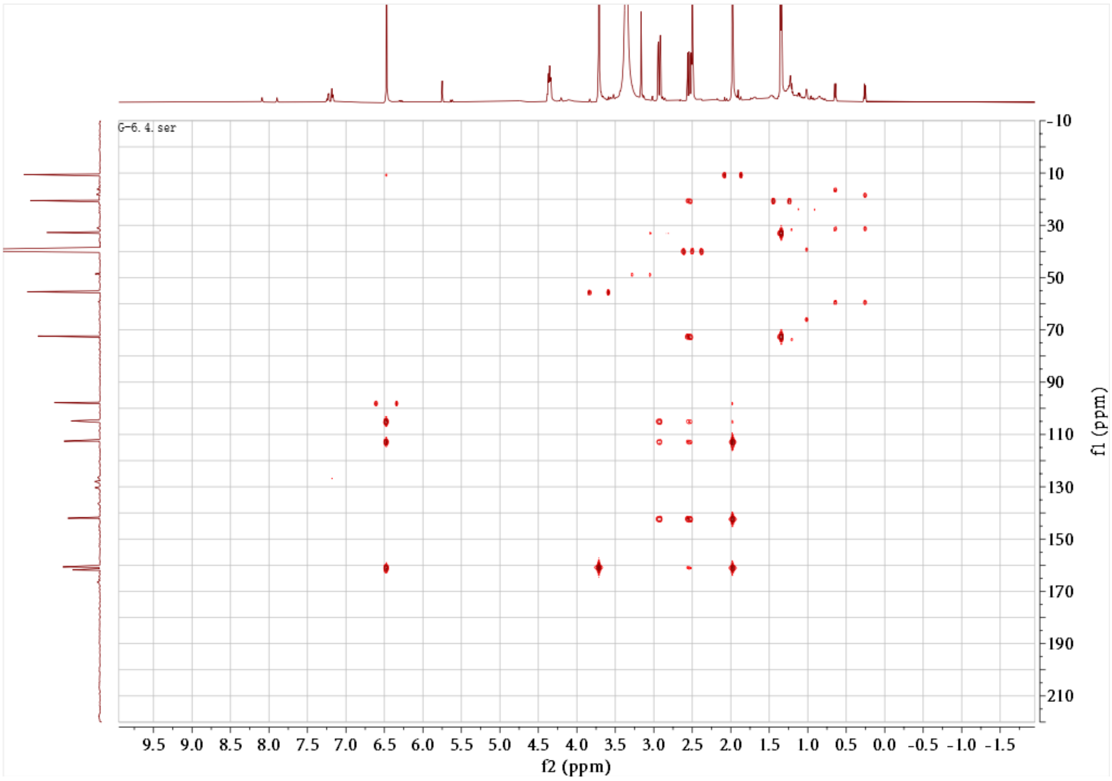

Figure S23. HMBC spectrum of compound **3** (DMSO)

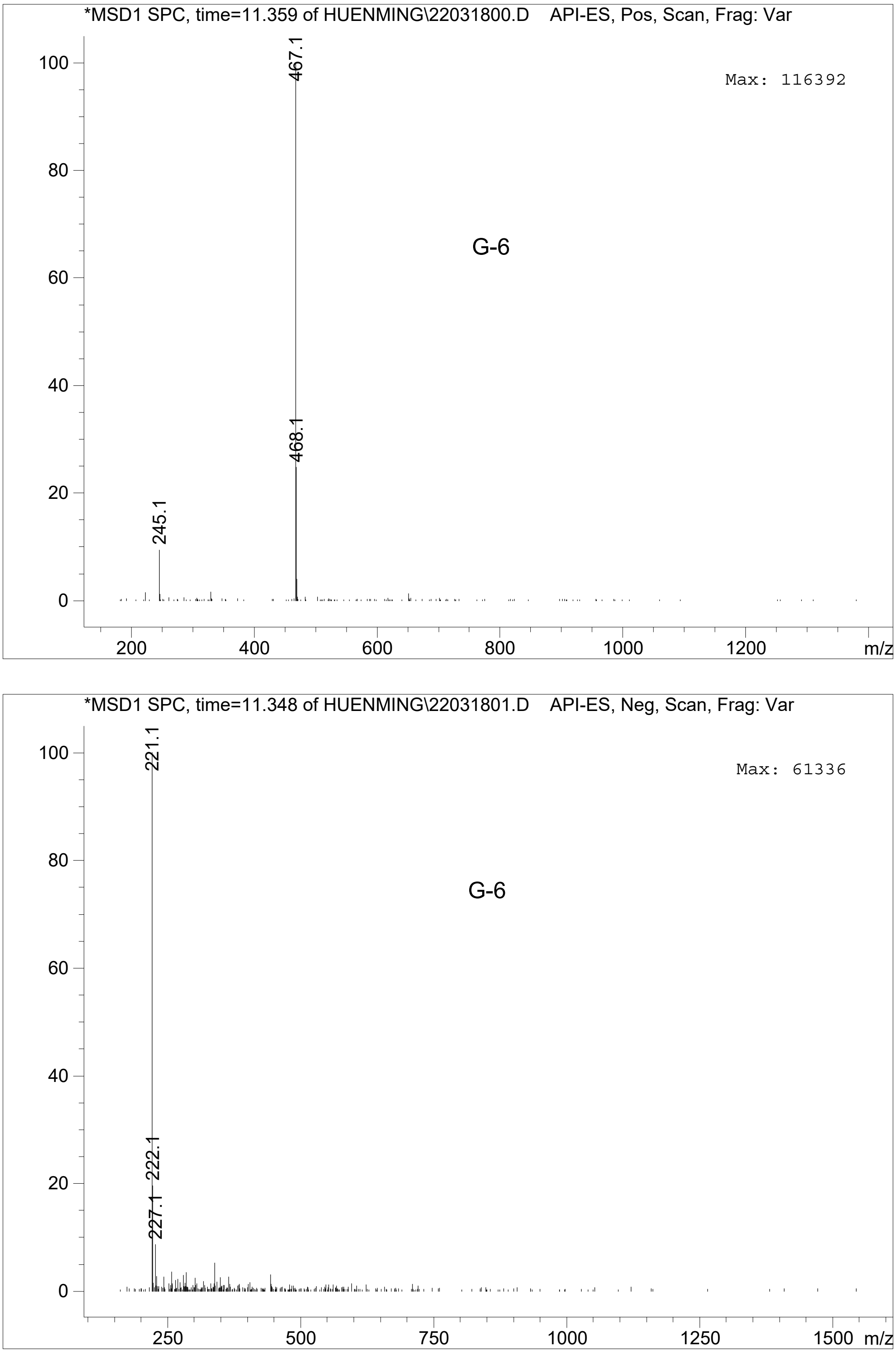

Figure S24. ESIMS of compound **3**

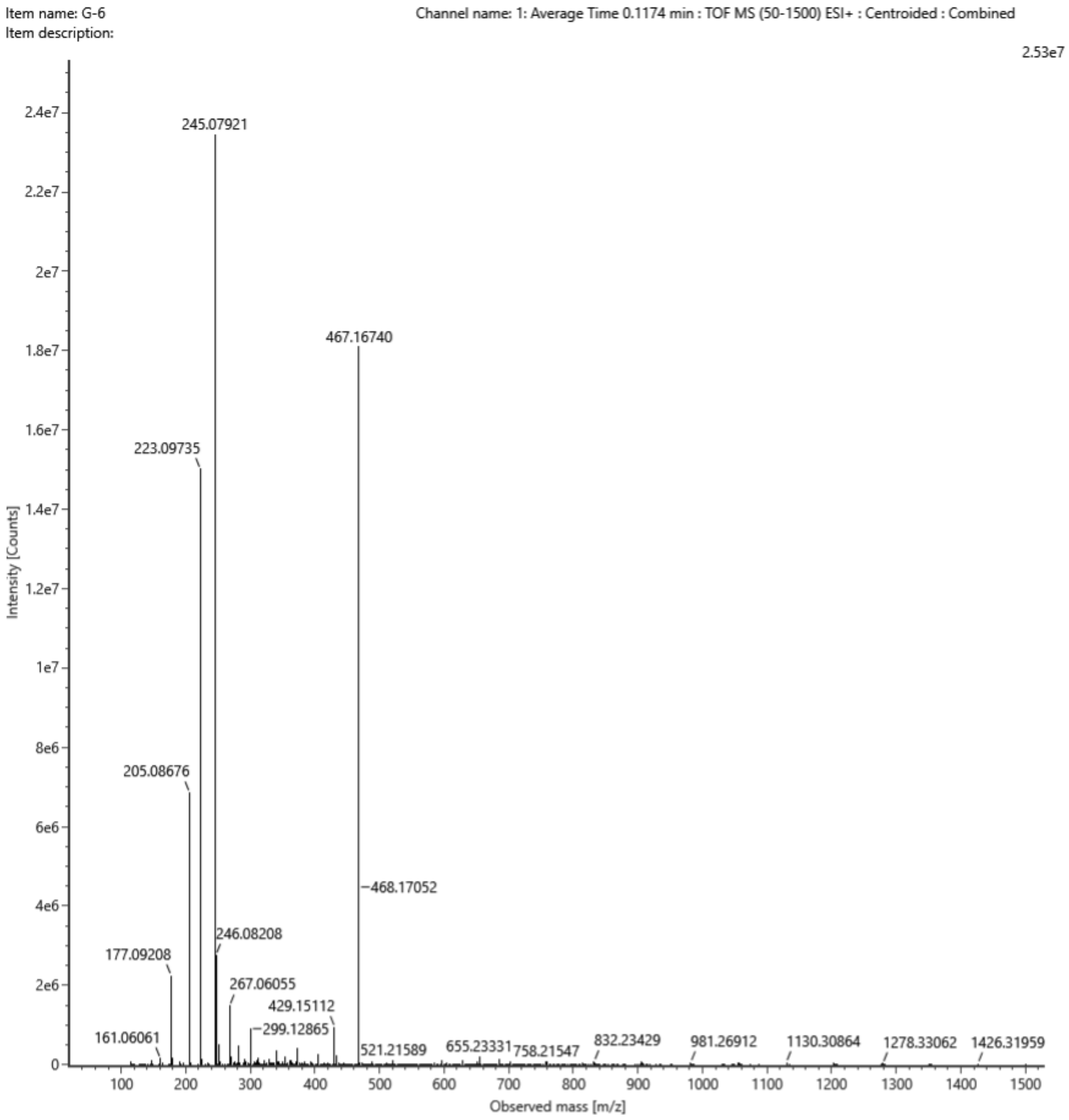

Add:Na+

Composition	i-FIT Confidence (%)	Predicted m/z	m/z error (PPM)
$C_{12}H_{14}O_4Na$	100.000000	245.078430	3.195714

Figure S25. HR-ESIMS of compound **3**

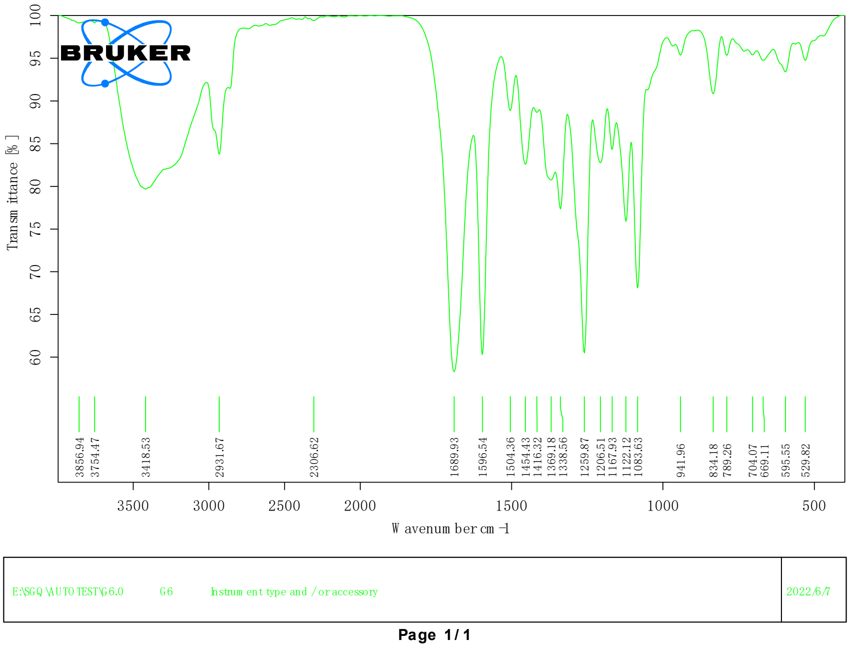

Figure S26. IR (KBr) spectrum of compound **3**

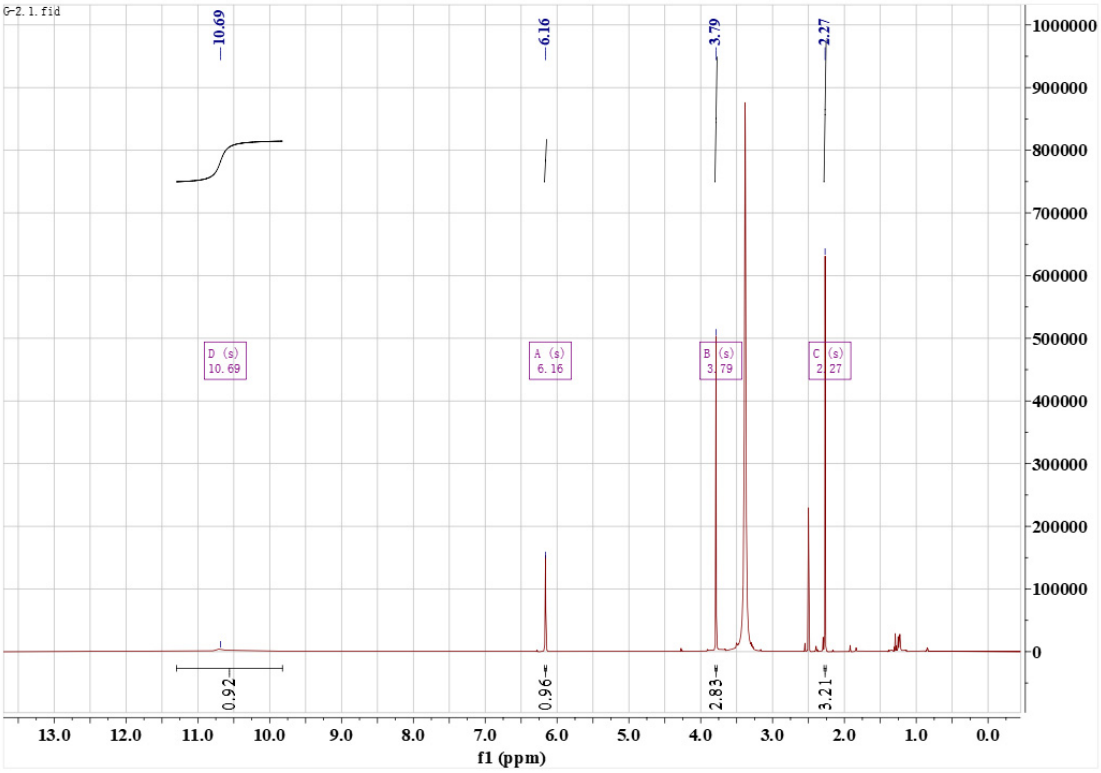

Figure S27. ^{1}H NMR spectrum of compound **4** (600 MHz, DMSO)

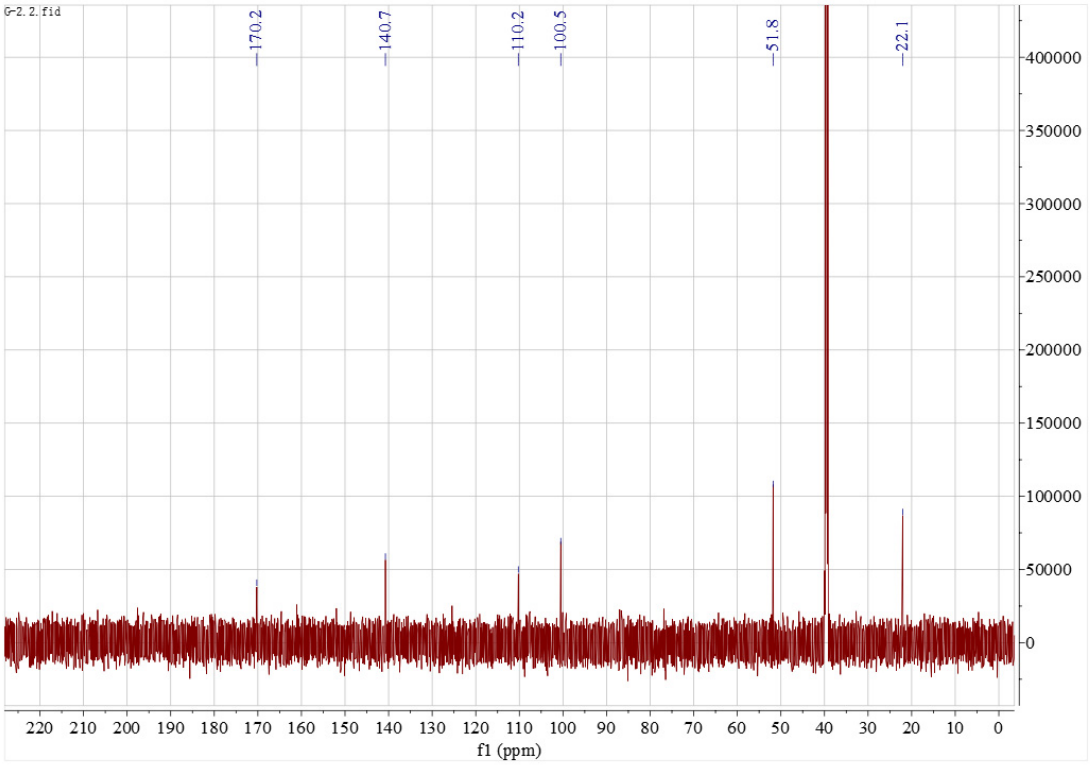

Figure S28. ^{13}C NMR spectrum of compound **4** (150 MHz, DMSO)

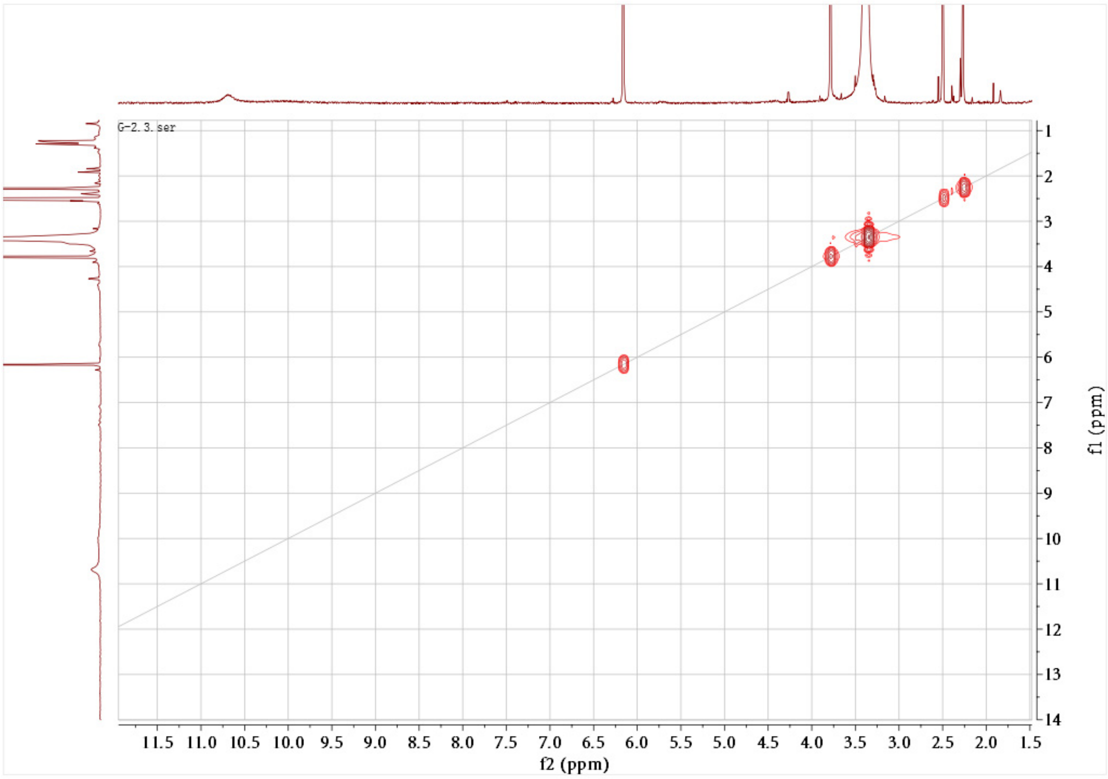

Figure S29. ^{1}H-^{1}H COSY spectrum of compound **4** (DMSO)

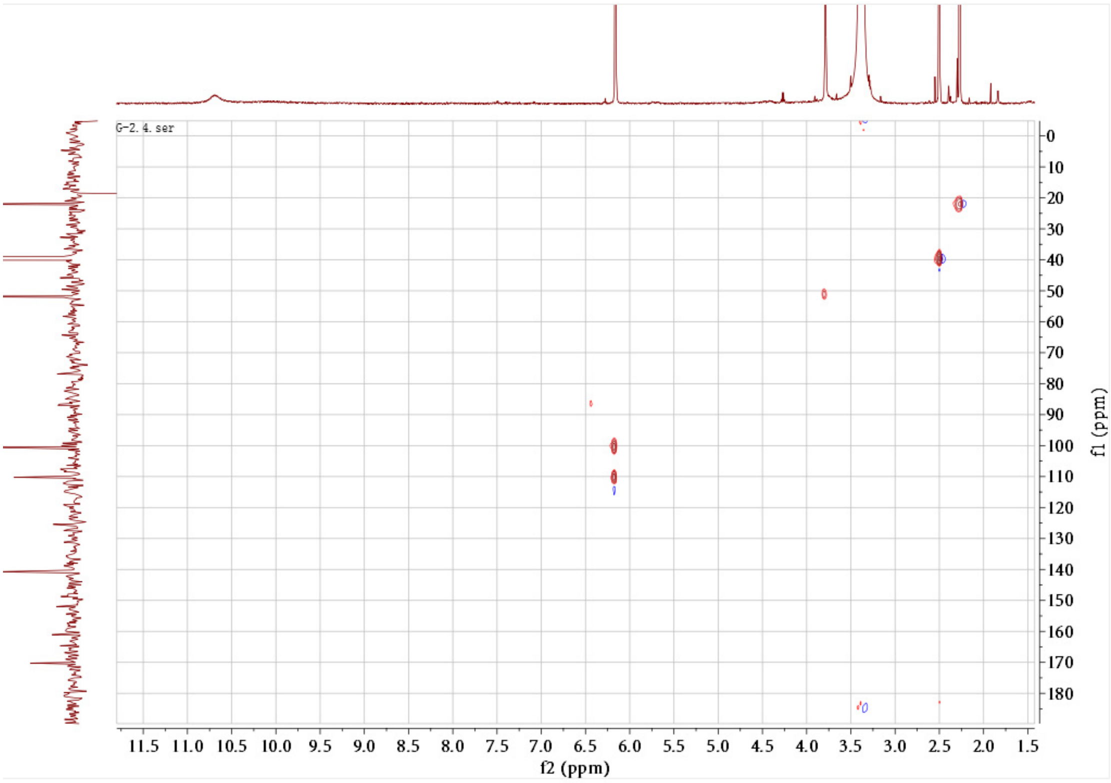

Figure S30. HSQC spectrum of compound **4** (DMSO)

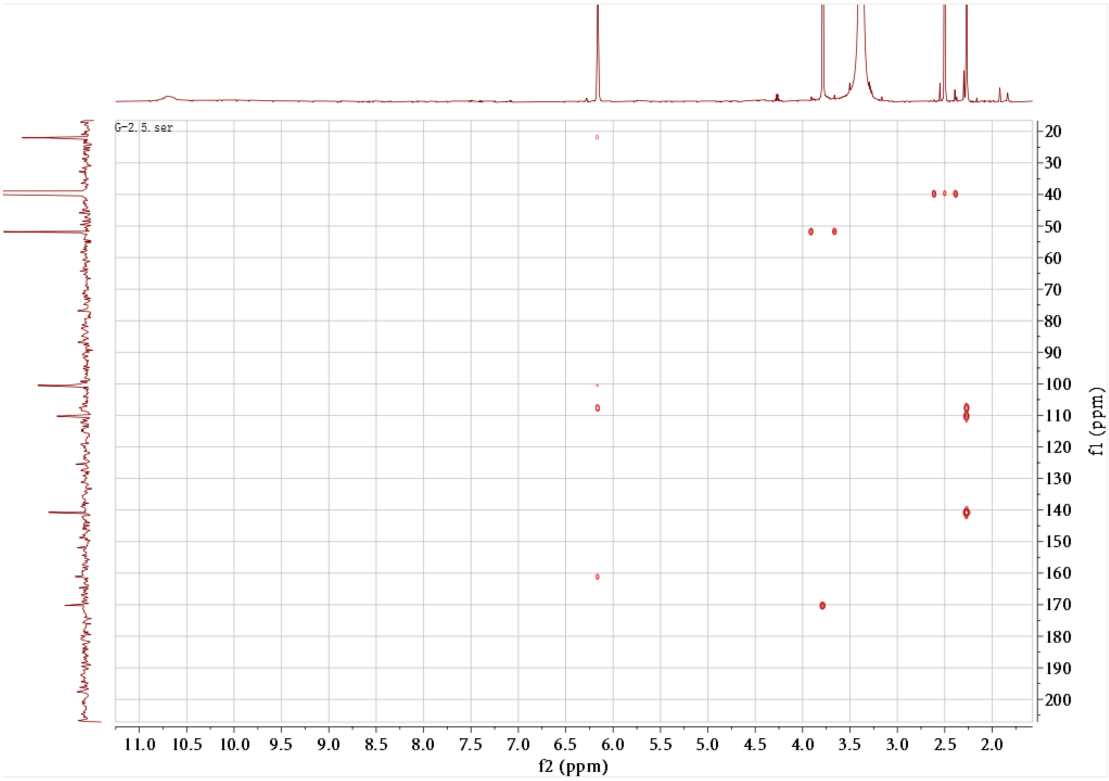

Figure S31. HMBC spectrum of compound **4** (DMSO)

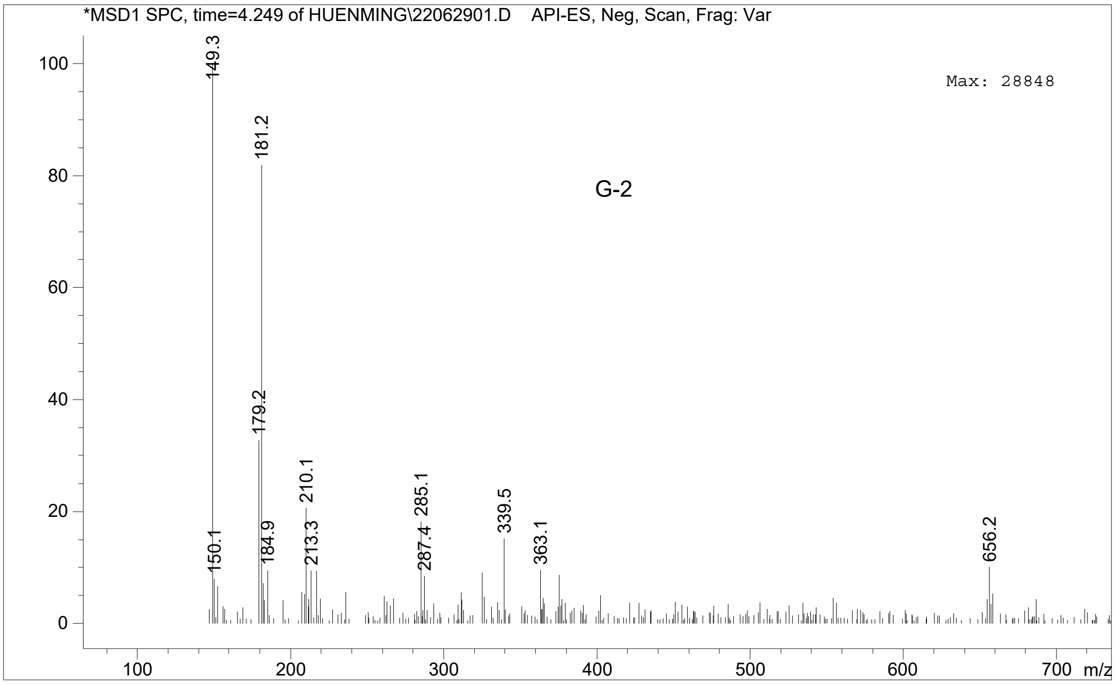

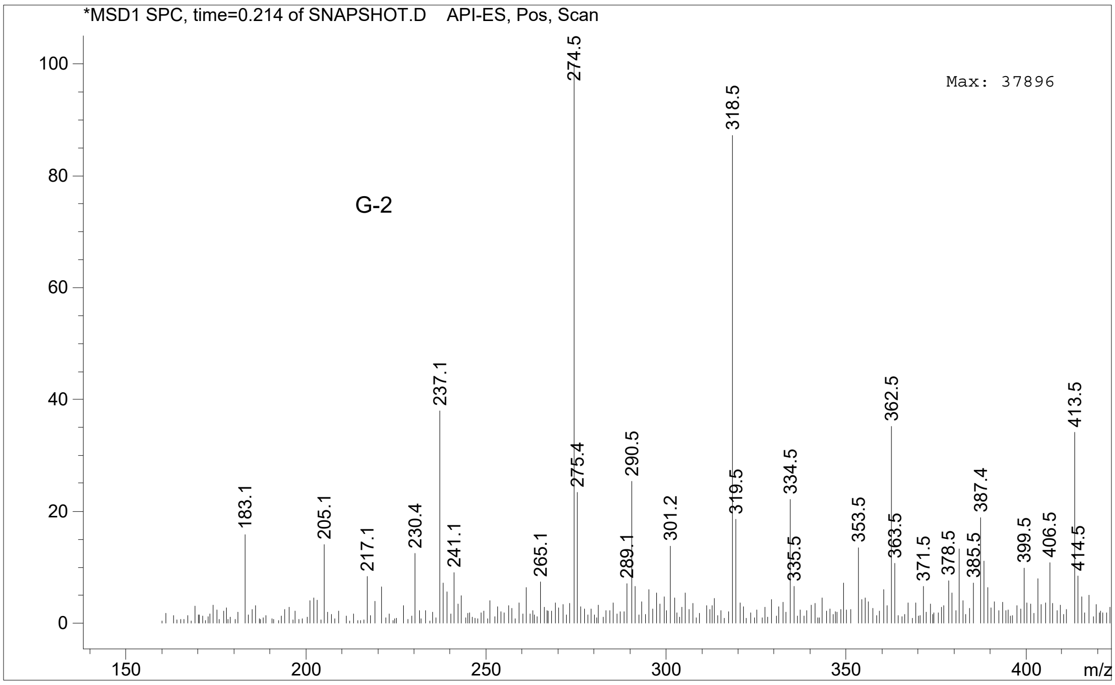

Figure S32. ESIMS of compound **4**

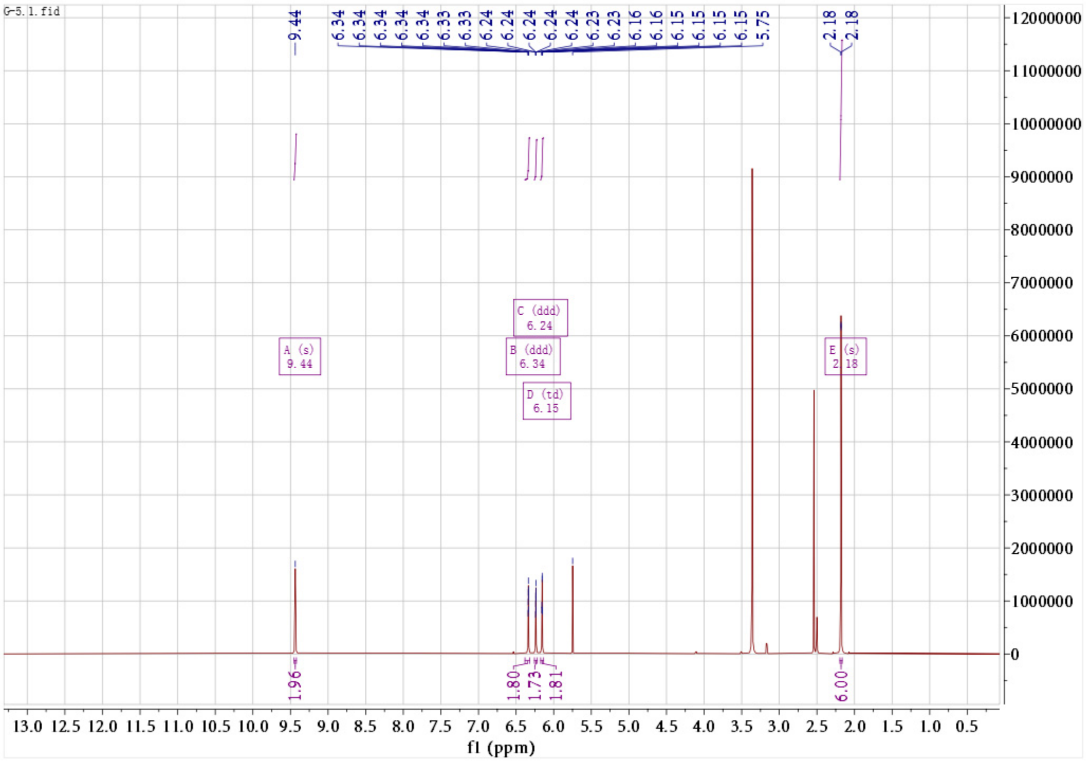

Figure S33. ^{1}H NMR spectrum of compound **5** (600 MHz, DMSO)

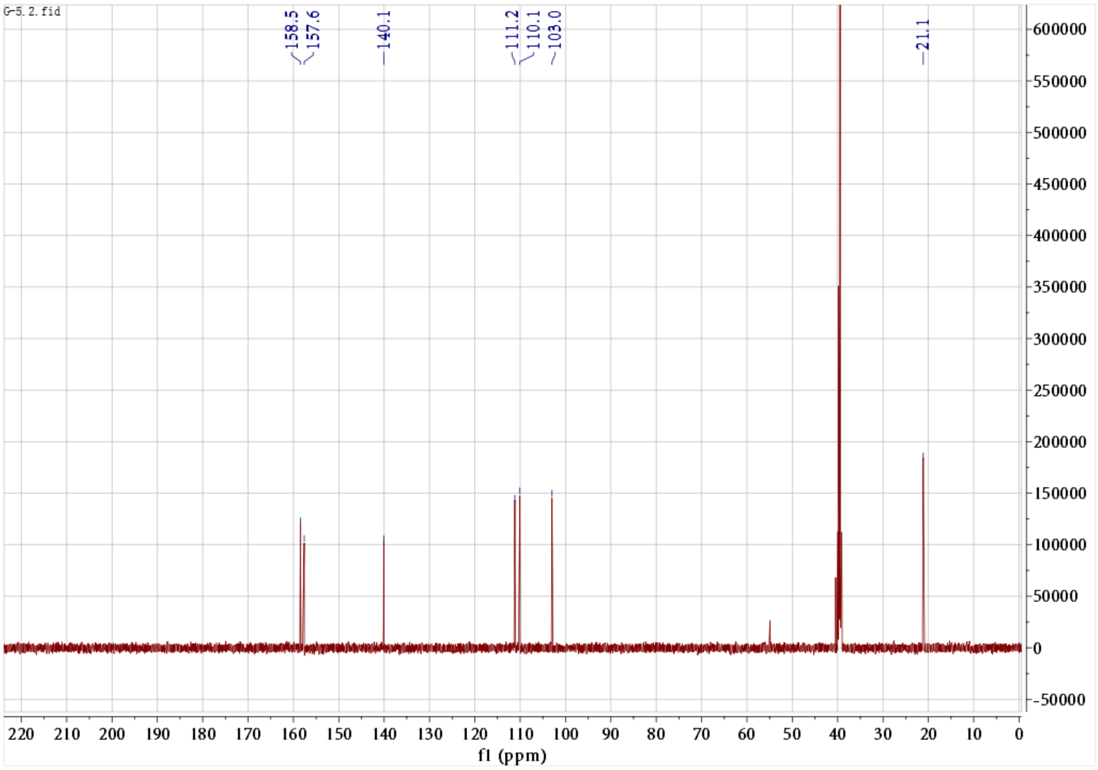

Figure S34. ^{13}C NMR spectrum of compound **5** (150 MHz, DMSO)

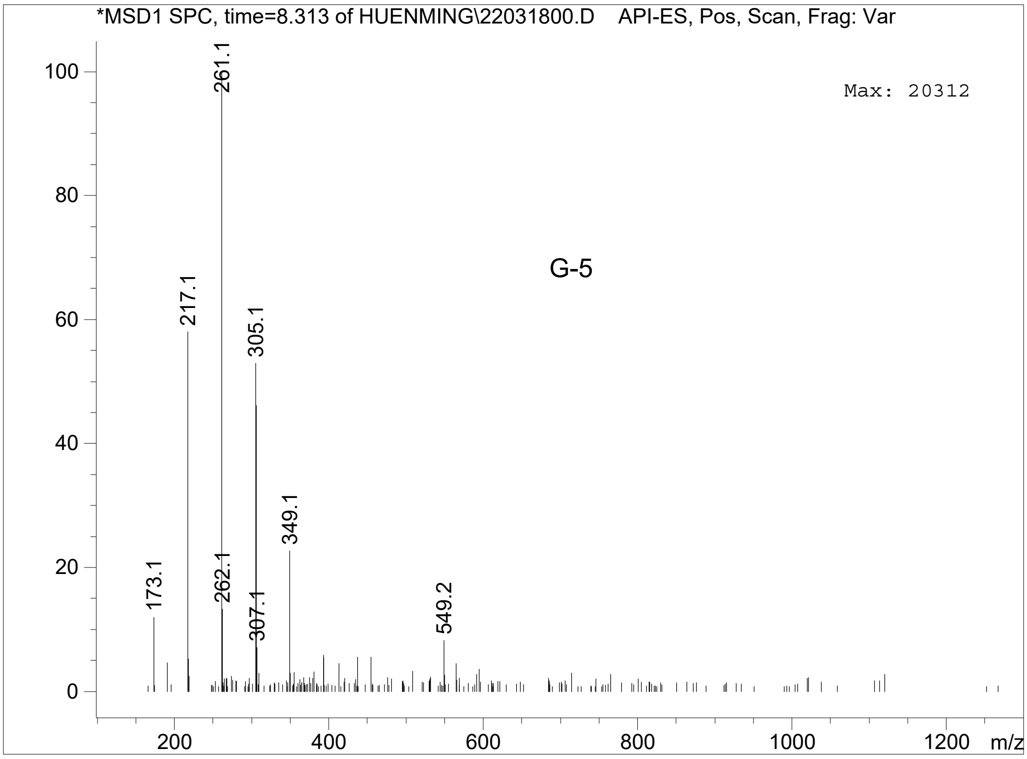

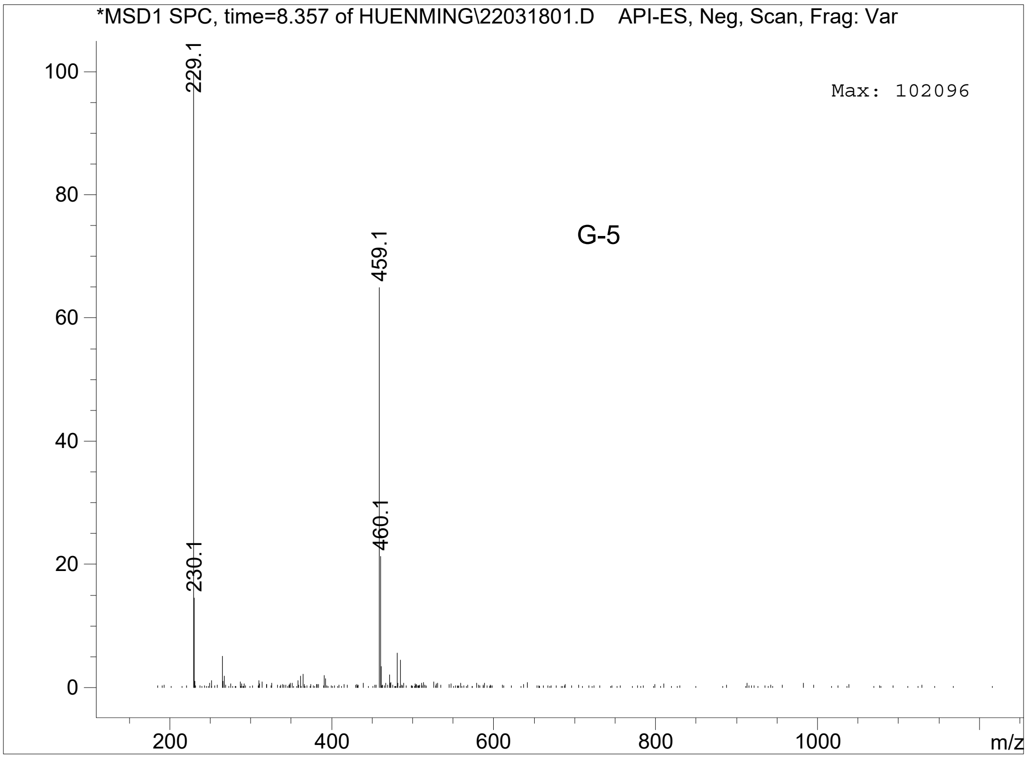

Figure S35. ESIMS of compound **5**

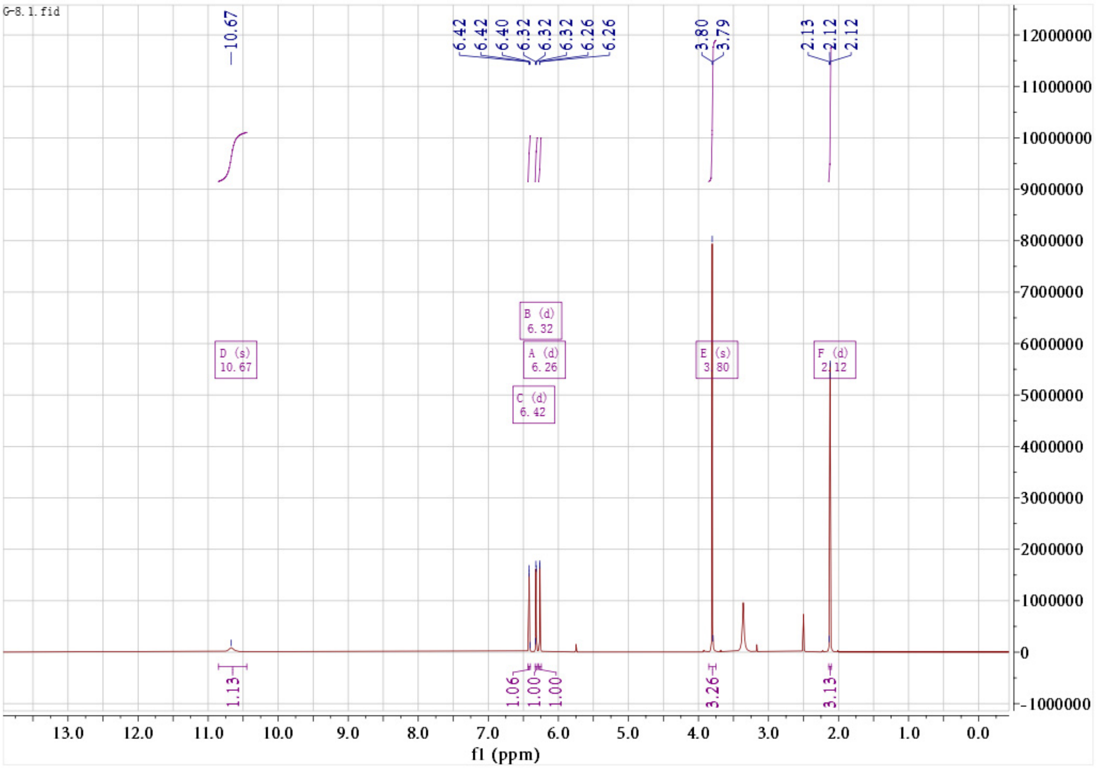

Figure S36. ^{1}H NMR spectrum of compound **6** (600 MHz, DMSO)

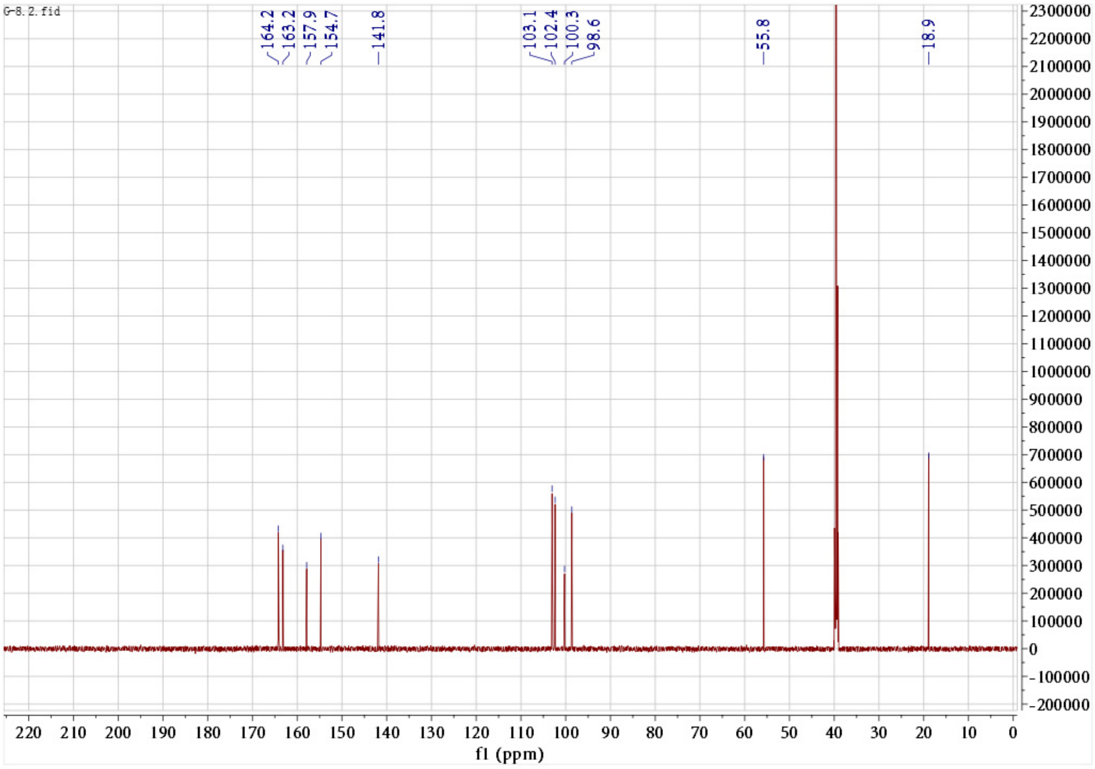

Figure S37. ^{13}C NMR spectrum of compound **6** (150 MHz, DMSO)

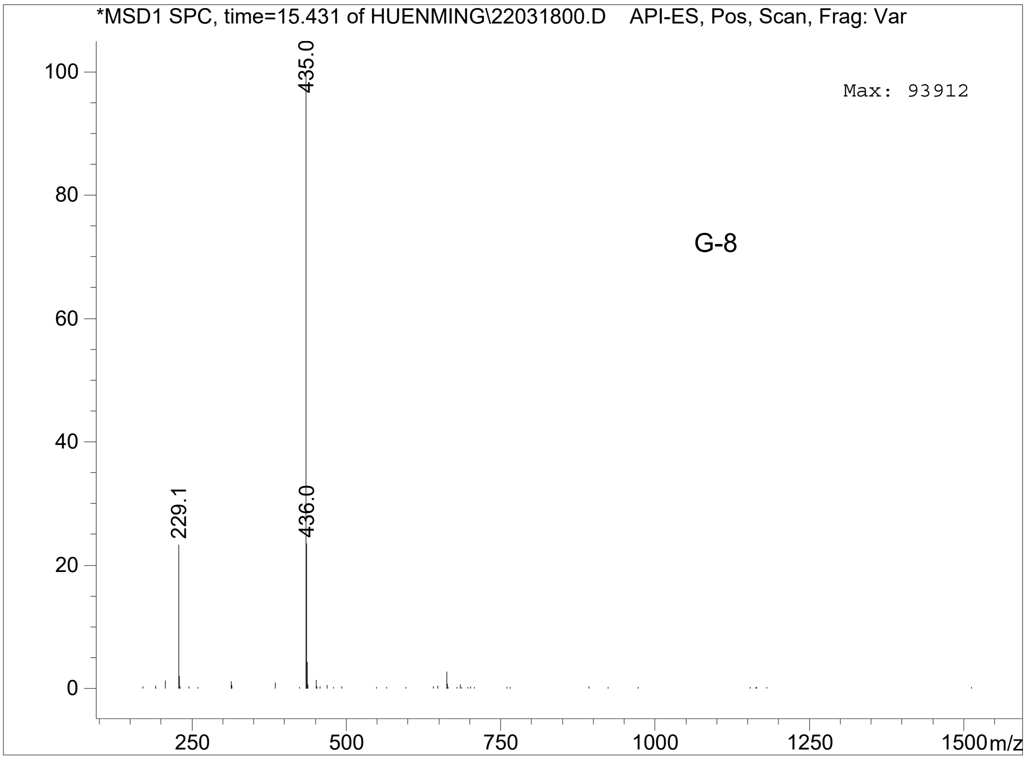

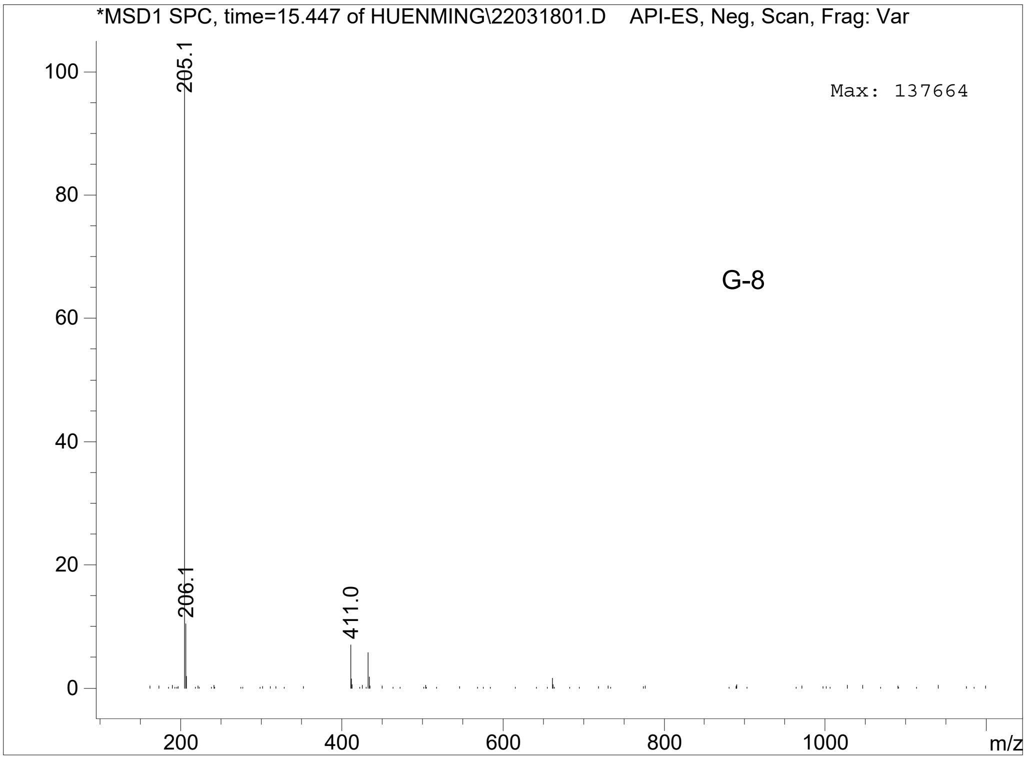

Figure S38. ESIMS of compound **6**

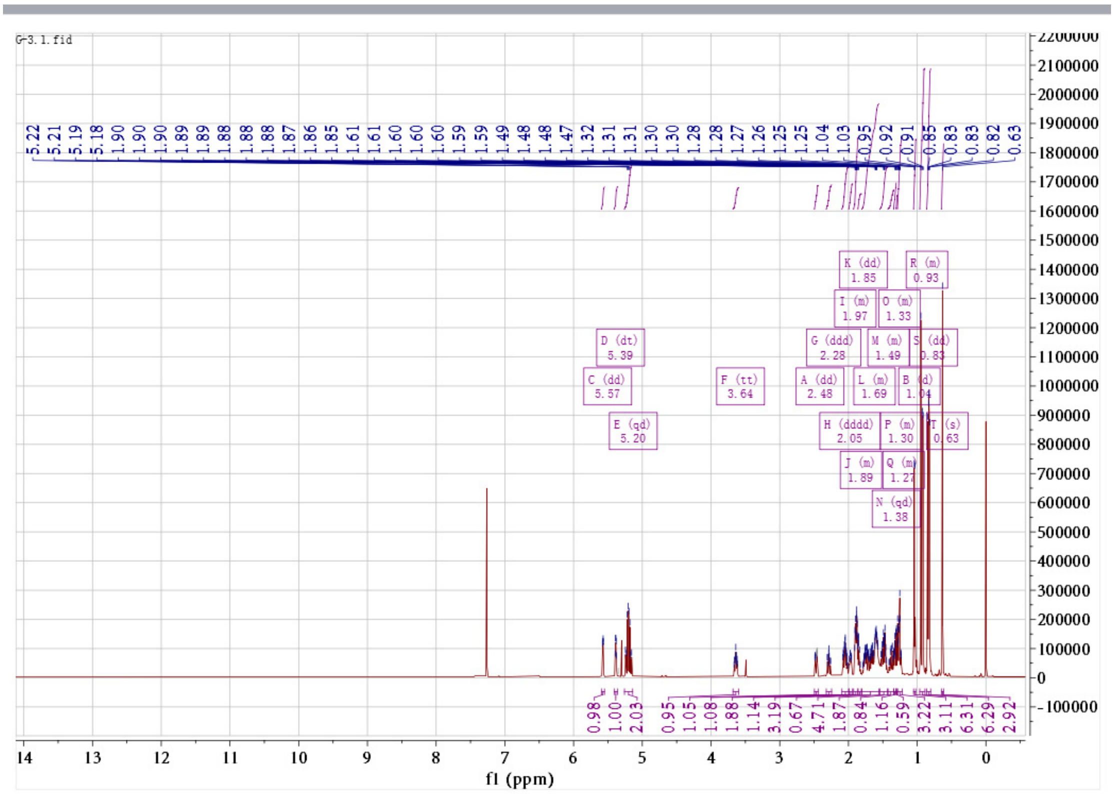

Figure S39. ^{1}H NMR spectrum of compound **7** (600 MHz, $CDCl_3$)

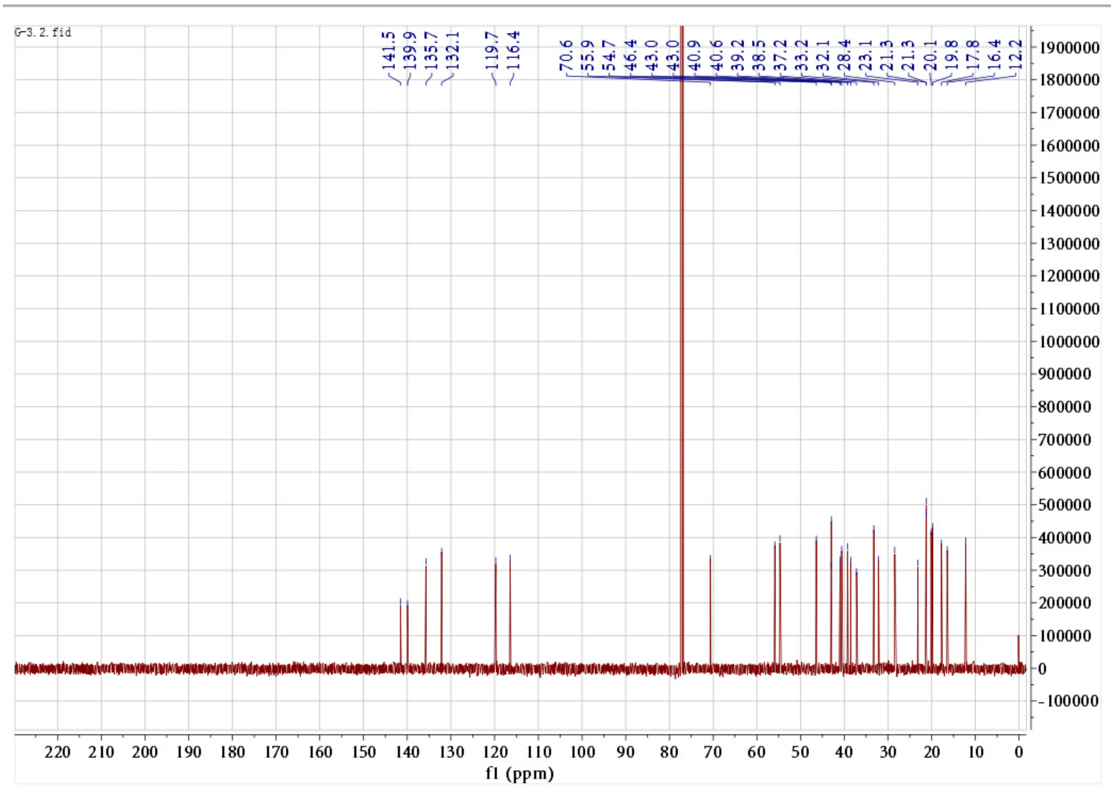

Figure S40. ^{13}C NMR spectrum of compound **7** (150 MHz, $CDCl_3$)

The conformations of the isomers of compounds **1** and **2** were generated by MM+ method embedded in HypeChem. Density functional theory calculations were performed with the Gaussian 16 package (Frisch et al., 2019). The remaining conformers were optimized at the B3LYP/6-31G(d) level in gas phase and the conformers within an energy window of 2 kcal/mol were kept. NMR shielding tensors were calculated with the GIAO method at the mPW1PW91/6- 311G(d,p) level with the IEFPCM solvent model in DMSO. The shielding constants were converted into chemical shifts by referencing to TMS at 0 ppm according to the formula δcal = σTMS–σcal, where the σTMS (the shielding constant of TMS) was calculated at the same level. DP4+ probability analysis was performed using the calculated NMR shielding tensors with DP4+ excel file (Grimblat et al., 2015). ECD spectra were calculated by the TDDFT methodology at the B3LYP/def2TZVP utilizing IEFPCM in methanol.

(1) Frisch,M. J., Trucks, G.W., Schlegel, H. B., Scuseria, G. E., Robb,M. A., Cheeseman, J. R., et al. (2019). Gaussian 16, Revision C.01. Wallingford CT: Gaussian, Inc.

(2) Grimblat, N., Zanardi, M. M., and Sarotti, A. M. (2015). Beyond DP4: An Improved Probability for the Stereochemical Assignment of Isomeric Compounds Using Quantum Chemical Calculations of NMR Shifts. J. Org. Chem. 80, 12526–12534. doi:10.1021/acs.joc.5b02396

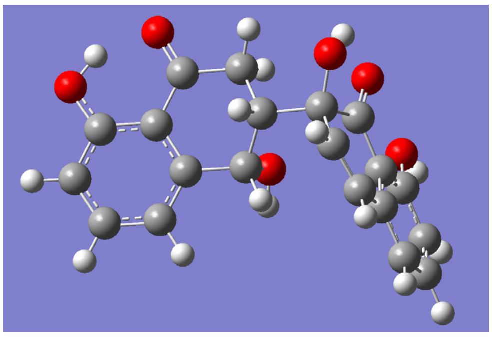

1a

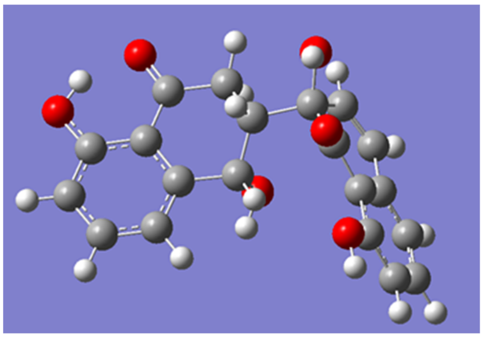

1b

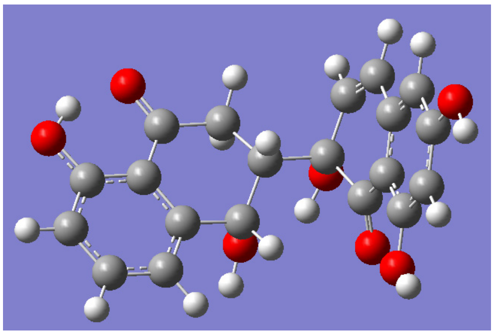

2a

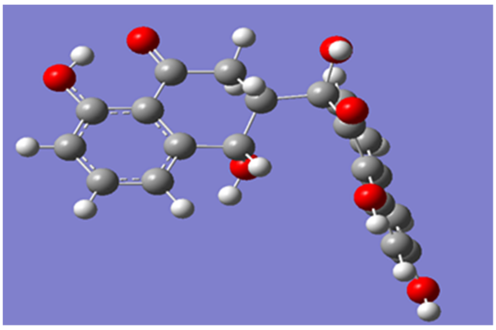

2b

Figure S41. Optimized geometries of isomers of **1** and **2** at B3LYP/6-31G(d) level.

Table. S1. Optimized cartesian coordinates of conformers of **1** at B3LYP/6-31G(d) level.

	**1a**			**1b**		
C	-9.4233	-4.1044	2.0695	-9.8397	-3.7055	0.9459
C	-9.924	-2.8891	2.5292	-10.2549	-2.4946	1.4941
C	-9.2079	-1.7107	2.3109	-9.3453	-1.4459	1.6407
C	-7.9805	-1.7324	1.624	-8.0049	-1.5958	1.2396
C	-7.4808	-2.9615	1.167	-7.5911	-2.8221	0.6936
C	-8.2081	-4.1404	1.3925	-8.516	-3.8679	0.5472
C	-7.2095	-0.45	1.434	-7.0273	-0.4665	1.4689
C	-6.2959	-0.4854	0.189	-5.8441	-0.5158	0.4859
C	-5.3978	-1.7337	0.2753	-5.1837	-1.9025	0.5471
C	-6.1832	-3.0097	0.4517	-6.1729	-3.0167	0.2974
C	-5.4441	0.8146	0.0319	-4.8066	0.6332	0.6643
C	-4.6049	0.7964	-1.217	-4.007	0.6295	1.9302
C	-4.8617	1.5133	-2.3166	-4.0968	1.5679	2.8799
C	-6.0257	2.3935	-2.3705	-5.035	2.6809	2.7423
C	-6.7878	2.6688	-1.2234	-5.7463	2.901	1.5514
C	-6.3508	2.075	0.0746	-5.479	2.0002	0.3952
C	-6.3638	2.9996	-3.5888	-5.2104	3.5585	3.821
C	-7.4552	3.8642	-3.6705	-6.0874	4.639	3.7209
C	-8.2241	4.1239	-2.5383	-6.8054	4.8482	2.5455
C	-7.8941	3.525	-1.3204	-6.6382	3.9795	1.4656
O	-8.6968	3.7613	-0.2409	-7.3871	4.1822	0.3418
O	-6.705	2.5603	1.15	-5.727	2.3335	-0.7654
O	-6.4257	-0.2314	2.6038	-6.5731	-0.5492	2.8138
O	-5.7527	-4.0935	0.0561	-5.8239	-4.103	-0.1667
O	-7.7835	-5.3751	0.9792	-8.1895	-5.0925	0.0282
H	-6.9462	-0.5901	-0.6927	-6.2627	-0.4198	-0.5291
O	-4.4999	0.9102	1.1004	-3.8184	0.4838	-0.3786
H	-9.9747	-5.0255	2.2407	-10.5433	-4.5266	0.8329
H	-10.872	-2.8622	3.0615	-11.2882	-2.3714	1.8104
H	-9.616	-0.7715	2.6821	-9.6889	-0.5092	2.0773
H	-7.9273	0.372	1.3395	-7.5584	0.4848	1.3518
H	-4.8296	-1.8448	-0.655	-4.4116	-1.9795	-0.2271
H	-4.683	-1.6693	1.1034	-4.7147	-2.0923	1.5184
H	-3.7176	0.1668	-1.2021	-3.3041	-0.1884	2.0669
H	-4.2052	1.4544	-3.1799	-3.4867	1.5021	3.7755
H	-5.7769	2.8078	-4.4853	-4.6629	3.4093	4.7497
H	-7.7079	4.3311	-4.6199	-6.2134	5.3145	4.5639
H	-9.0818	4.7855	-2.6236	-7.4959	5.6853	2.4908
H	-9.2114	4.5714	-0.3887	-7.7493	5.0831	0.3448
H	-7.03	-0.2489	3.3684	-7.2649	-0.9657	3.3573
H	-6.9105	-5.2623	0.5382	-7.2319	-5.0784	-0.201
H	-5.035	0.9173	1.9238	-4.0714	1.1093	-1.0888

Table S2. The results of the DP4+ analysis of **1**

Functional		Solvent?		Basis Set		Type of Data	
mPW1PW91		PCM		6-31+G(d,p)		Shielding Tensors	
		DP4+	0.00%	100.00%	-	-	-
Nuclei	sp2?	xperimenta	Isomer 1	Isomer 2	Isomer 3	Isomer 4	Isomer 5
C		205.1	-7.8	-7.9			
C		32.8	159.0	160.2			
C		49.2	147.1	139.5			
C		65.9	123.4	125.5			
C	x	145.7	53.5	51.1			
C	x	119.7	81.2	80.7			
C	x	137.1	59.0	59.8			
C	x	117.1	79.7	79.9			
C	x	161.4	35.2	35.0			
C	x	114.7	82.3	82.7			
C	x	206.4	-7.5	-7.0			
C		73.5	111.6	117.1			
C	x	136.3	59.9	62.9			
C	x	125.7	69.7	68.3			
C	x	137.6	57.0	54.9			
C	x	118.9	78.1	78.2			
C	x	137.8	61.6	60.2			
C	x	117.1	82.1	82.4			
C	x	161.5	41.8	41.4			
C	x	113.2	79.5	82.3			

Table. S3. Optimized cartesian coordinates of conformers of 2 at B3LYP/6-31G(d) level

	**2a**			**2b**		
C	-9.3964	-4.235	2.2546	-9.9012	-3.712	0.8603
C	-9.9058	-3.0171	2.6976	-10.2848	-2.5119	1.4533
C	-9.1999	-1.8363	2.4598	-9.3426	-1.5033	1.6617
C	-7.9743	-1.8585	1.7697	-8.0006	-1.6828	1.2783
C	-7.4657	-3.0901	1.3298	-7.6189	-2.8987	0.6873
C	-8.1827	-4.2713	1.5749	-8.5764	-3.9038	0.4788
C	-7.2143	-0.5729	1.558	-6.9899	-0.5985	1.5718
C	-6.3061	-0.6191	0.3095	-5.7876	-0.6494	0.612
C	-5.3966	-1.8582	0.4111	-5.1788	-2.0609	0.6273
C	-6.1695	-3.1383	0.6118	-6.201	-3.1264	0.3089
C	-5.4666	0.6859	0.1286	-4.7129	0.4519	0.8598
C	-4.6307	0.6544	-1.1222	-3.9381	0.3612	2.1377
C	-4.8929	1.3546	-2.2312	-4.0144	1.2557	3.1299
C	-6.0595	2.2305	-2.2951	-4.9137	2.4041	3.0313
C	-6.8239	2.515	-1.152	-5.5947	2.7059	1.8408
C	-6.3845	1.9388	0.1534	-5.3325	1.8547	0.646
C	-6.3977	2.8249	-3.5177	-5.0813	3.2333	4.1472
C	-7.4877	3.6848	-3.5987	-5.9187	4.3417	4.0759
C	-8.2653	3.9601	-2.4805	-6.609	4.6435	2.908
C	-7.933	3.3691	-1.2586	-6.4474	3.8199	1.7916
O	-8.7387	3.6071	-0.1808	-7.1666	4.0989	0.6639
O	-6.7447	2.4367	1.2211	-5.5368	2.261	-0.4994
O	-6.4274	-0.3291	2.7208	-6.5677	-0.7468	2.9213
O	-5.7298	-4.2247	0.2342	-5.8811	-4.2031	-0.1969
O	-7.7491	-5.5086	1.1791	-8.2822	-5.1157	-0.0873
H	-6.9591	-0.7431	-0.5677	-6.1806	-0.4955	-0.4061
O	-4.5208	0.8082	1.1928	-3.7093	0.3067	-0.1685
O	-7.7636	4.2377	-4.8138	-6.0385	5.1116	5.1945
H	-9.9399	-5.1579	2.4409	-10.6303	-4.5022	0.6989
H	-10.8526	-2.9901	3.232	-11.3192	-2.3661	1.7562
H	-9.6146	-0.8951	2.8181	-9.6621	-0.5747	2.1326
H	-7.9396	0.2413	1.4546	-7.4862	0.3743	1.4811
H	-4.8321	-1.9792	-0.5201	-4.3926	-2.1335	-0.133
H	-4.6784	-1.7741	1.2345	-4.7392	-2.3087	1.5993
H	-3.7405	0.0291	-1.1013	-3.2651	-0.4854	2.2471
H	-4.2385	1.2864	-3.0958	-3.4237	1.1273	4.0319
H	-5.8133	2.6299	-4.4144	-4.5603	3.0249	5.079
H	-9.1275	4.6136	-2.5583	-7.2739	5.4996	2.8685
H	-9.2182	4.4423	-0.3052	-7.4573	5.0253	0.6732
H	-7.0221	-0.3704	3.492	-7.2795	-1.1694	3.4334
H	-6.8781	-5.3956	0.7342	-7.3205	-5.1256	-0.2984
H	-5.0529	0.8186	2.0181	-3.8488	1.041	-0.8007
H	-8.5218	4.8397	-4.7323	-6.6322	5.8587	5.0126

Table S4. The results of the DP4^{+} analysis of **2**

	A	B	C	D	E	F	G	H	
1	Functional		Solvent?		Basis Set		Type of Data		
2	mPW1PW91		PCM		6-31G(d)		Shielding Tensors		
3									
12			DP4+	3.13%	96.87%	–	–	–	
14	Nuclei	sp2?	xperimenta	Isomer 1	Isomer 2	Isomer 3	Isomer 4	Isomer 5	Isor
15	C		205	-7.7	-8.0				
16	C		33.2	159.6	160.2				
17	C		48.8	147.2	139.7				
18	C		65.7	124.3	125.2				
19	C	x	146	53.3	51.2				
20	C	x	119.5	80.2	80.7				
21	C	x	137.2	58.4	59.8				
22	C	x	117	79.5	79.8				
23	C	x	161.4	35.4	34.7				
24	C	x	114.7	82.5	82.8				
25	C	x	203.4	-3.7	-3.5				
26	C		73.6	112.6	117.6				
27	C	x	136.8	59.7	61.3				
28	C	x	125.3	70.7	68.4				
29	C	x	139.6	55.6	52.8				
30	C	x	107.9	90.7	90.1				
31	C	x	166.2	36.4	35.5				
32	C	x	101.5	96.9	97.7				
33	C	x	164.8	40.1	38.8				
34	C	x	106.7	88.0	88.0				
